# Illuminating subduction zone rheological properties in the wake of a giant earthquake

**DOI:** 10.1126/sciadv.aax6720

**Published:** 2019-12-18

**Authors:** Jonathan R. Weiss, Qiang Qiu, Sylvain Barbot, Tim J. Wright, James H. Foster, Alexander Saunders, Benjamin A. Brooks, Michael Bevis, Eric Kendrick, Todd L. Ericksen, Jonathan Avery, Robert Smalley, Sergio R. Cimbaro, Luis E. Lenzano, Jorge Barón, Juan Carlos Báez, Arturo Echalar

**Affiliations:** 1 COMET, School of Earth and Environment, University of Leeds, Leeds, UK.; 2 Institute of Geosciences, University of Potsdam, Potsdam, Germany.; 3 Earth Observatory of Singapore, Nanyang Technological University, Singapore.; 4 Asian School of the Environment, Nanyang Technological University, Singapore.; 5 Department of Earth Sciences, University of Southern California, Los Angeles, CA, USA.; 6 Hawaii Institute of Geophysics and Planetology, University of Hawaii at Manoa, Honolulu, HI, USA.; 7 U.S. Geological Survey Earthquake Science Center, Menlo Park, CA, USA.; 8 School of Earth Sciences, Ohio State University, Columbus, OH, USA.; 9 Center for Earthquake Research and Information, University of Memphis, Memphis, TN, USA.; 10 Dirección de Geodesia, Instituto Geográfico Nacional, Buenos Aires, Argentina.; 11 International Center for Earth Sciences, Universidad Nacional de Cuyo, Mendoza, Argentina.; 12 Centro Sismológico Nacional, Universidad de Chile, Santiago, Chile.; 13 Instituto Geográfico Militar, La Paz, Bolivia.

## Abstract

Deformation associated with plate convergence at subduction zones is accommodated by a complex system involving fault slip and viscoelastic flow. These processes have proven difficult to disentangle. The 2010 *M*_w_ 8.8 Maule earthquake occurred close to the Chilean coast within a dense network of continuously recording Global Positioning System stations, which provide a comprehensive history of surface strain. We use these data to assemble a detailed picture of a structurally controlled megathrust fault frictional patchwork and the three-dimensional rheological and time-dependent viscosity structure of the lower crust and upper mantle, all of which control the relative importance of afterslip and viscoelastic relaxation during postseismic deformation. These results enhance our understanding of subduction dynamics including the interplay of localized and distributed deformation during the subduction zone earthquake cycle.

## Teaser

We invert postseismic geodetic data to reveal subduction zone mechanical properties following a megathrust earthquake in Chile.

The largest earthquakes occur on the gently dipping fault planes that comprise the shallow portions of subduction zones at convergent tectonic plate margins (*1*, *2*). The stress change imparted by these megathrust earthquakes induces postseismic slip on the megathrust fault (i.e., afterslip) and transient flow in the lower crust (LC) and upper mantle (i.e., viscoelastic relaxation) that can persist for years to decades (*1*, *3*). The associated postseismic surface displacements can be used to infer the frictional properties of the fault system (*4*), to probe the rheological structure of the surrounding crust and upper mantle (*5*, *6*), and to better understand the evolution of stress during the seismic cycle (*4*, *7*–*10*).

Only four giant [*M*_w_ (moment magnitude) ≥8.5] subduction earthquakes have occurred in the era of satellite geodesy: the 2004 *M*_w_ 9.2 Sumatra-Andaman and the 2005 *M*_w_ 8.6 Nias-Simeulue, both in Sumatra, Indonesia; the 2010 *M*_w_ 8.8 Maule, Chile; and the 2011 *M*_w_ 9.0 Tohoku-Oki, Japan events (*7*, *11*, *12*). Analyses of these and other great (*M*_w_ ≥8.0) earthquakes have yielded useful, yet often conflicting conclusions regarding the inferred rheological properties. This is due to the difficulty of resolving the relative contributions of the various postseismic deformation mechanisms (*1*, *3*, *4*, *13*, *14*) and particularly because of the mechanical coupling between afterslip and viscoelastic flow (*9*, *15*, *16*).

**Fig. 1:**
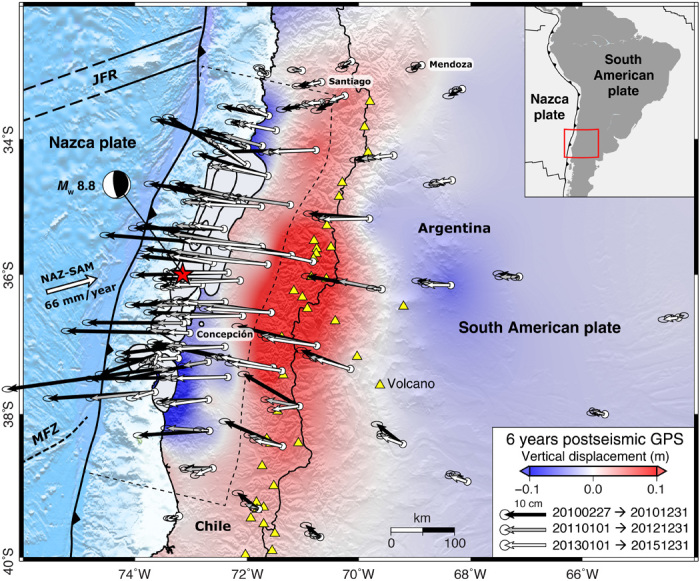
Evolution of postseismic surface displacements for the first 6 years following the 27 February 2010 *M*_w_ 8.8 Maule earthquake in Chile from CGPS data. Background colors show interpolated CGPS-derived vertical motion for the entire observation period overlain on hill-shaded topography. The earthquake centroid location (red star) and focal mechanism, the outline of the megathrust from the model (dashed black polygon), and 5-m coseismic slip contours (black outline with gray filling) (*24*), which we use to calculate the initial coseismic stress changes in our inversion, are also shown. JFR, Juan Fernández Ridge; MFZ, Mocha Fracture Zone.

The 27 February 2010 *M*_w_ 8.8 Maule earthquake was the largest to occur along the Nazca–South America tectonic plate boundary since the 1960 *M*_w_ 9.5 Valdivia earthquake (*17*, *18*). In contrast to the Sumatran earthquakes that were observed with a sparse geodetic network and the Tohoku-Oki earthquake that occurred far out to sea, near- and far-field Maule surface deformation histories have been captured by a dense regional network of continuously operating Global Positioning System (CGPS) sites (Fig. 1 and fig. S1). The network includes coastal stations located a few tens of kilometers from the offshore region of maximum coseismic slip as well as sites distributed across the spine of the high Andes, backarc, and adjacent foreland basin (Fig. 1 and fig. S1). Researchers have used the associated data to examine a wide range of earthquake cycle–related phenomena (*11*, *17*, *19*–*25*). Recent studies have focused on GPS stations located >300 km from the epicenter in an attempt to isolate the role of viscoelastic relaxation (*26*, *27*), although this process may also dominate proximal postseismic deformation particularly after large (*M*_w_ ≥8.0) earthquakes (*13*, *15*).

**Fig. 2:**
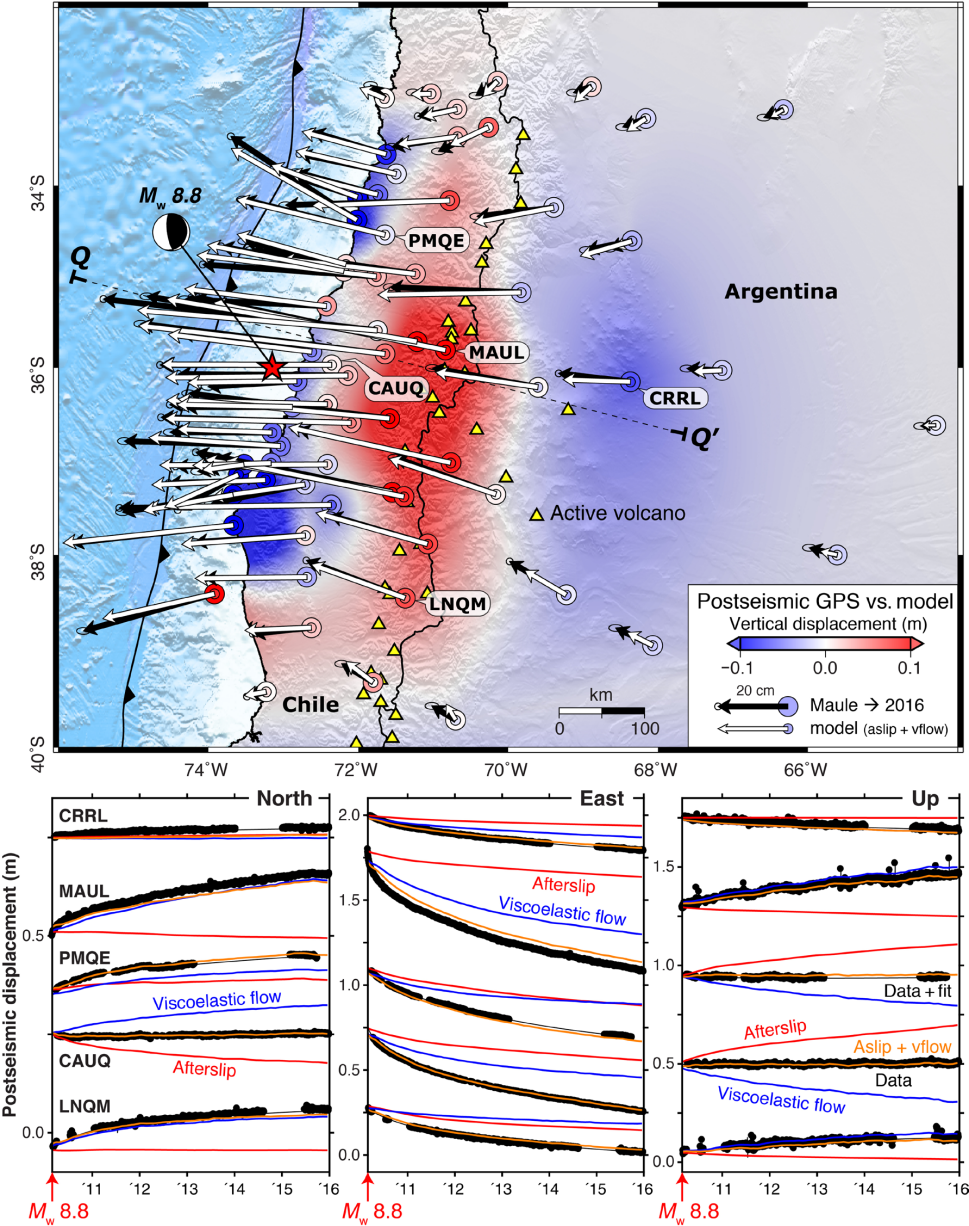
Observed and modeled postseismic surface displacements. (**Top**) Cumulative horizontal CGPS displacements (black vectors) and model results reflecting the combined contribution of afterslip on the megathrust and ductile flow in the LC and upper mantle (white vectors). Cumulative vertical CGPS and model displacements are shown as nested large and small colored circles, respectively. Background colors show interpolated model vertical motions overlain on hill-shaded topography for direct comparison with Fig. 1. (**Bottom**) Postseismic displacement time series for example sites. Black circles and lines are the daily positions and empirical fits. Red, blue, and orange curves are afterslip, viscoelastic flow, and total model predictions, respectively (see fig. S2 for all sites). *Q*-*Q*′ marks the location of the profiles shown in Fig. 5.

We assemble time-dependent horizontal and vertical motions from 6 years of CGPS data following the Maule earthquake (Figs. 1 and 2, and fig. S2) and invert the geodetic time series for the kinematics of afterslip on the megathrust and viscous strain in the LC and upper mantle without invoking a priori assumptions regarding the constitutive behavior (*7*, *8*, *28*). Our approach combines the standard method of using Green’s functions to map slip on a fault patch to surface displacements with solutions for the stress and surface displacements caused by distributed anelastic strain of deformable volumes placed at depth (*28*). The Maule event offers an ideal opportunity to exploit this novel imaging technique, and we provide a new synoptic view of the megathrust fault, lithosphere-asthenosphere rheological structure, and the evolution of stress and strain during the subduction zone earthquake cycle across this highly seismogenic tectonic plate boundary.

## Maule postseismic displacement field

The CPGS-derived horizontal and vertical Maule postseismic displacement fields are shown in Fig. 1. Full descriptions can be found elsewhere (*20*, *23*, *24*, *26*, *27*), but it is worth noting that many postseismic modeling studies exclude vertical GPS data because they are often complicated and characterized by higher uncertainty than the horizontal data despite the notion that the evolution of postseismic vertical displacements, particularly for large dip-slip earthquakes, is critical for distinguishing between afterslip and viscoelastic relaxation (*3*, *7*, *14*). This is also the case in Chile, where post-Maule vertical displacements change sign from uplift to subsidence as a function of distance from the earthquake (Figs. 1 and 2, and fig. S2) (*23*, *24*, *26*). The large-scale near- and mid-field postseismic vertical displacement pattern, although much lower in absolute magnitude, is generally in the opposite sense to the coseismic vertical motion, which was characterized by coastal uplift and broad subsidence across the high Andes (*11*, *24*, *26*).

## Afterslip and viscoelastic flow modeling approach and results

**Fig. 3:**
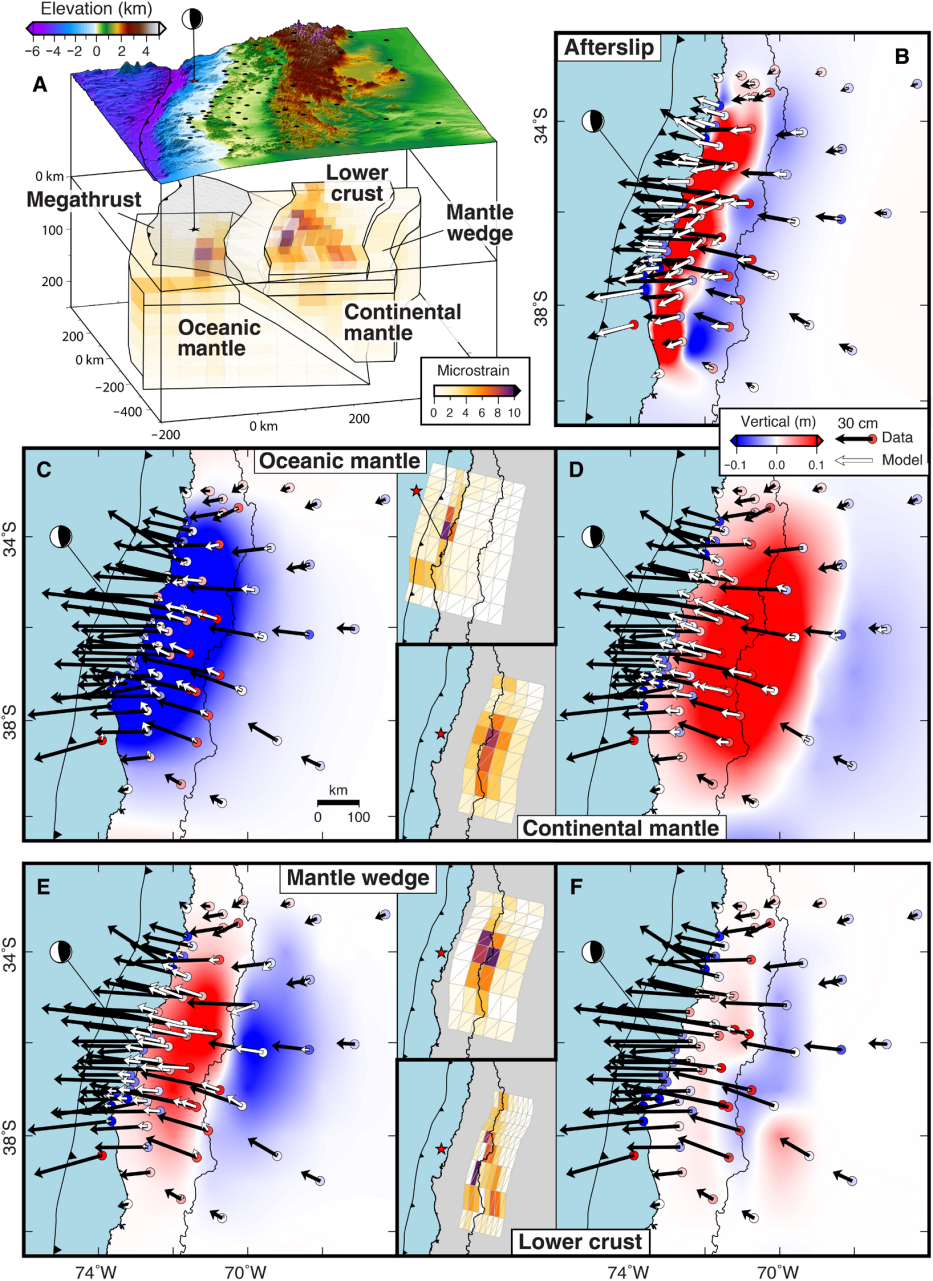
Inversion model geometry, surface displacements, and strain contributions from different portions of the lithosphere-asthenosphere system. (**A**) Perspective view of regional topography and bathymetry, CGPS site distribution (black circles), semitransparent discretized megathrust, and semitransparent polyhedral volumes colored by cumulative total deviatoric viscous strain (second invariant). (**B**) Afterslip contribution to the surface displacement field with total horizontal (black vectors) and vertical (colored circles) CGPS displacements. The model horizontal and vertical afterslip contributions are shown with white vectors and background colors, respectively. (**C** to **F**) Model surface displacements due to viscoelastic flow in the lithosphere-asthenosphere system. Vectors and background colors are the same as in (B). Insets show the map view, total cumulative strain with the same color scale as in (A).

We discretize the subduction zone interface using a network of triangular boundary elements that extend along strike for ~400 km and from the trench to a depth of ~80 km (Fig. 3). This is large enough to encompass the region of coseismic slip and to invert for the kinematics of afterslip on surrounding portions of the megathrust fault. To represent bulk deformation, we adopt a curvilinear mesh to match the curvature of the plate interface and capture the role of different regions in accommodating the viscoelastic relaxation of earthquake-induced stresses. The model constitutes a consistent representation of the lithosphere-asthenosphere system and allows for the possibility of arbitrary variations in properties between portions of the oceanic mantle (OM) beneath the megathrust fault, the continental mantle (CM) at the down-dip termination of the discretized megathrust fault, the mantle wedge (MW) above the CM, and the LC directly beneath the volcanic arc (Fig. 3 and fig. S3). Authors often refer to all of the upper mantle above the slab as the MW, while we assign this label to the lithospheric mantle located between the LC and the CM. We calculate the initial coseismic stress changes using a slip model (*24*) and simultaneously invert the postseismic surface displacement time series at each CGPS site for localized afterslip on the megathrust and distributed deformation in the ductile regions using a Kalman filter [Supplementary Materials; (*7*)]. Model sensitivity tests (fig. S5) show that deformation contributions from all these regions are necessary to satisfactorily explain the postseismic surface observations and the evolution of stress in the upper mantle.

Our preferred model (Supplementary Materials) matches the observed horizontal and vertical displacement magnitudes and azimuths, although we slightly underestimate total displacements in a few scattered locations (Fig. 2). We reproduce the large-scale patterns of vertical motion including the localized subsidence north and south of the earthquake, the broad transition to uplift across the high Andes straddling the Chile-Argentina border, and the subsidence in western Argentina. Our estimated strain magnitudes are also consistent with previous postseismic studies (*3*) and three-dimensional forward models of fault slip and viscoelastic flow during the earthquake cycle (Supplementary Materials).

The model also explains the temporal evolution of postseismic deformation (Fig. 2 and fig. S2). Near the volcanic arc, the contributions of afterslip and viscoelastic flow exhibit opposite senses of vertical motion. This is most obvious for CGPS sites PMQE and CAUQ, located less than 40 km from the coastline, which experienced several tens of centimeters of afterslip-induced uplift. Viscoelastic flow, however, produces comparable amounts of subsidence, resulting in no substantial net vertical deformation. In contrast, most of the postseismic horizontal and vertical motions for the high Andes CGPS site MAUL are dominated by viscoelastic mantle flow.

Exploiting the linearity of the kinematics of deformation, we can separate the fingerprints of afterslip and viscoelastic flow stemming from different parts of the lithosphere-asthenosphere system (Fig. 3). Afterslip-related deformation is most pronounced in the near- to mid-field and is responsible for the localized coastal subsidence (Figs. 1 and 3), which rapidly transitions eastward to coastline-parallel uplift and again to subsidence midway between the coast and the Chile-Argentina border before diminishing across the backarc. Afterslip also accounts for ≥50% of the horizontal motion between the coast and the high Andes, but the associated horizontal displacement vectors above the zone of afterslip-induced uplift all point to the southwest, oblique to the general westward trend of the total displacement vectors.

Viscoelastic flow in the OM produces a broad zone of subsidence surrounding the rupture that gradually decreases in magnitude with distance from the earthquake (Fig. 3). OM strain is greatest just beneath the earthquake, decreasing with distance and depth from this location. The OM contribution to the horizontal displacements is less pronounced than from afterslip, resulting in a few centimeters of landward motion along the coast but up to a few tens of centimeters of seaward displacement toward the earthquake with peak values in central Chile that systematically decrease across the high Andes and backarc. This landward-to-seaward change in direction occurs over a few tens of kilometers. Viscoelastic flow of the OM is the only known process that produces landward postseismic displacements at coastal sites (*9*, *13*, *14*).

Viscoelastic flow in the CM results in a broad zone of uplift that extends from the coastline to the backarc with the largest displacements (horizontal and vertical) just west of the high Andes. CM strain concentrates directly beneath the volcanic arc. Together, the OM and CM are responsible for the broad vertical deformation signal and change from near-field subsidence to uplift across the high Andes.

MW flow results in a vertical deformation pattern characterized by uplift extending from the coastline to the volcanic arc with a transition to subsidence across the Chile-Argentina border and further east. This pattern contributes significantly to the total horizontal displacement field particularly in central Chile across-strike from the earthquake. As with the CM, MW strain concentrates beneath the volcanic arc. In contrast, LC flow minimally affects the postseismic deformation field contributing only about a centimeter of horizontal and vertical motion. The decrease in CGPS site density moving inland combined with the smaller size of the associated volumes affects our ability to resolve LC strain. However, those that are well resolved indicate relatively small amounts of distributed lower crustal strain (Fig. 3 and fig. S6).

## Frictional afterslip on the subduction megathrust

**Fig. 4:**
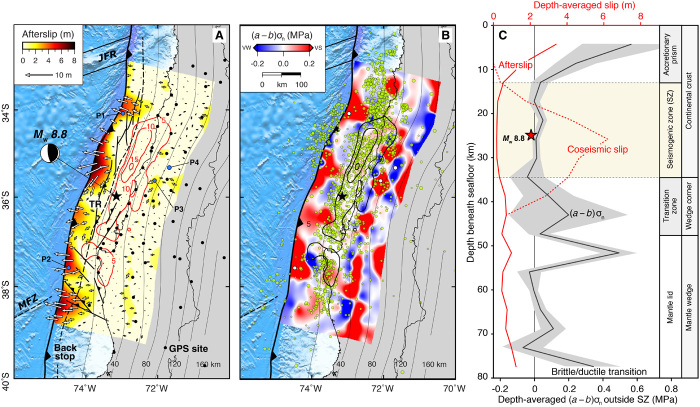
Derived afterslip and frictional parameter on the megathrust fault. (**A**) Six years of cumulative afterslip on the Maule megathrust fault, coseismic slip contours (*24*) in 5-m increments, the depth of the subducting slab in 20-km increments, the north-south trending thrust ridge (TR), additional offshore, upper-plate faults, and the approximate location of the backstop (*34*, *38*). P1 to P4 correspond to example locations where system trajectories used in the frictional parameter calculation are shown in fig. S7. (**B**) Rate-and-state frictional parameter on the megathrust fault calculated between 20 days and 12 months after the earthquake. Red regions are velocity strengthening (VS), blue regions are velocity weakening (VW) or stable weakening, and light blue/red regions with values close to 0 are velocity neutral. Light green circles are the first week of aftershocks. (**C**) Depth-averaged afterslip, scaled rate-and-state frictional parameter with minimum/maximum, and coseismic slip model. Inferred correlations with structural position are labeled on the far right. Note that the afterslip curve is well defined in areas of high afterslip, primarily surrounding the seismogenic zone as our inversion penalizes slip from occurring in regions that slipped coseismically.

We find up to 8 m of afterslip in shallow patches with a few small, isolated deep patches with no more than ~2 to 3 m of postseismic slip (Fig. 4). Our results differ from those of previous studies (*11*, *19*, *23*, *24*), primarily in that the majority of our afterslip occurs at shallow depths, up-dip from and partially surrounding the coseismic rupture with very little down-dip as is often the case with models that incorporate both afterslip and viscoelastic flow (*7*, *13*, *15*, *29*). This significant difference arises because our inversion includes the vertical component of the CGPS measurements, which captures coseismic uplift followed by postseismic subsidence along the coast and requires slip on different portions of the megathrust, specifically shallow afterslip. The absence of significant afterslip below the coseismic rupture suggests a shallow brittle-ductile transition or a semibrittle region (*30*).

Shallow afterslip explains both the measured horizontal displacements near the fault zone and coastal subsidence. In Sumatra, shallow afterslip up-dip of coseismic rupture has been observed following the 2005 *M*_w_ 8.7 Nias-Simeulue (*4*) and the 2007 *M*_w_ 8.4 Bengkulu (*29*) earthquakes, implying that the unconsolidated, shallow accretionary prism (AP) sediments may be velocity strengthening (VS) (*31*). However, the frictional properties of shallow megathrusts are likely more complex than previously assumed as evidenced by recent earthquakes, such as the Tohoku-Oki event, that propagated to the trench (*2*, *12*, *32*) and the discovery of slow-slip, tremors, and very low-frequency earthquakes above the seismogenic zone at various subduction zones (*33*).

To explore megathrust fault frictional properties, we assume that the temporal evolution of afterslip is controlled by steady-state rate-and-state friction

$$(a-b)\sigma_{n}=\frac{d\Delta \text{CFS}}{d\;\text{log}\;(V)}$$

where *a* and *b* are the rate-and-state frictional parameters (*a − b* defines the fault behavior), σ_n_ is the effective normal stress, ΔCFS is the coseismic Coulomb stress change on the creeping fault element, and *V* is the steady-state sliding velocity (*4*, *10*, *31*). ΔCFS and *V* can be determined from our inversion, allowing (*a − b*)σ_n_ to be estimated in locations where afterslip occurs (fig. S7). In this framework, if *a − b* > 0, then the material is considered VS, and primarily stable sliding (i.e., creep and afterslip) occurs. VS regions inhibit earthquake nucleation and rupture propagation. In contrast, if *a − b* < 0, then the material is velocity weakening (VW) and potentially seismic (i.e., frictional instabilities may nucleate). Although VW is required for earthquake nucleation, this condition is insufficient to fully predict slip behavior because a VW patch can be stable (i.e., no fast earthquake nucleation), exhibit complex characteristics including slow-slip and very low-frequency events, and participate in coseismic slip that initiates elsewhere. We refer to these regions as stable weakening. In contrast, we refer to fault patches that are both VW and unstable (e.g., most of the seismogenic zone) as unstable weakening (UW).

Our frictional property estimates (Fig. 4) are compatible with seismogenic faults in different tectonic settings (*4*, *10*) and support the view that Maule megathrust friction is variable (*24*, *25*, *34*). Coseismic slip and the majority of aftershocks occurred primarily across a UW region, which contrasts the notion that VS regions produce aftershocks in partially coupled fault zones (*4*, *10*). Note that the afterslip-based friction estimates are well defined primarily outside of the seismogenic region. The area surrounding the coseismic rupture, particularly up-dip of where most of the afterslip occurred, is predominantly VS. A notable exception is a VW patch that matches the region of maximum slip from a model that places significant shallow coseismic fault slip near the trench at the approximate latitude of an outer-rise aftershock cluster (Figs. 4 and 6, and fig. S7) (*35*). This spatial correlation supports the notion that coseismic rupture may have reached the trench in some places and triggered the outer-rise aftershock sequence (*36*). The variations in frictional behavior seem to allow coseismic rupture to occasionally propagate to the trench (*2*, *32*, *36*), other times allowing only shallow afterslip (*4*, *29*).

These results permit a more in-depth comparison with geological and geophysical evidence pointing toward structural control of Maule coseismic rupture. Despite the initial suggestion that coseismic slip was confined to the area between the Mocha Fracture Zone in the south and the Juan Fernández Ridge in the north (Figs. 1 and 4) (*37*), more recent work has shown that the rupture extent was controlled by splay faults that cut the upper plate and intersect the megathrust rather than just the aforementioned bathymetric features (*34*, *38*). The spatial correlation between the up-dip limit of significant coseismic slip from most of the studies (*11*, *17*, *24*) and the north-south trending thrust ridge (TR; Fig. 4), composed of splays that mark the approximate boundary (i.e., backstop) between the active, frontal, and paleoaccretionary prisms (*38*, *39*), is readily apparent. High-resolution bathymetric and seismic reflection and refraction data also reveal that the TR corresponds to the submarine shelf break, providing an indication of AP width, which varies from ~20 to 50 km across the coseismic rupture region (*37*, *39*). With the exception of the outer rise events, the TR also coincides with the up-dip limit of intense aftershock activity (Fig. 4).

Therefore, significant shallow afterslip was confined to a portion of the plate interface underlying the AP, and the frictional behavior is controlled by the hanging wall rheology (Fig. 4). Near-trench, along-strike differences in afterslip magnitude and friction parameter could represent changes in AP width over very short distances, which may be reflected in the sinuosity of the shelf break imaged in high-resolution seafloor bathymetry data (*39*). This interpretation allows for the possibility of coseismic slip to reach the trench (*35*) where the AP is absent or poorly developed.

The shallow megathrust region beneath the AP is often assumed VS, thus impeding dynamic rupture (*31*), but this assumption has been called into question in light of recent shallow slow-slip events and tsunami earthquakes (*2*, *33*). Our results for Maule provide a detailed picture revealing shallow megathrust frictional properties with significant along- and across-strike variability that can presumably result in drastically different shallow slip behavior over short distances (Fig. 4). The up-dip boundary of the seismogenic zone may be controlled by variations in both frictional properties and structure, marked by a distributed network of faults in the AP.

## Evolution of effective viscosity

**Fig. 5:**
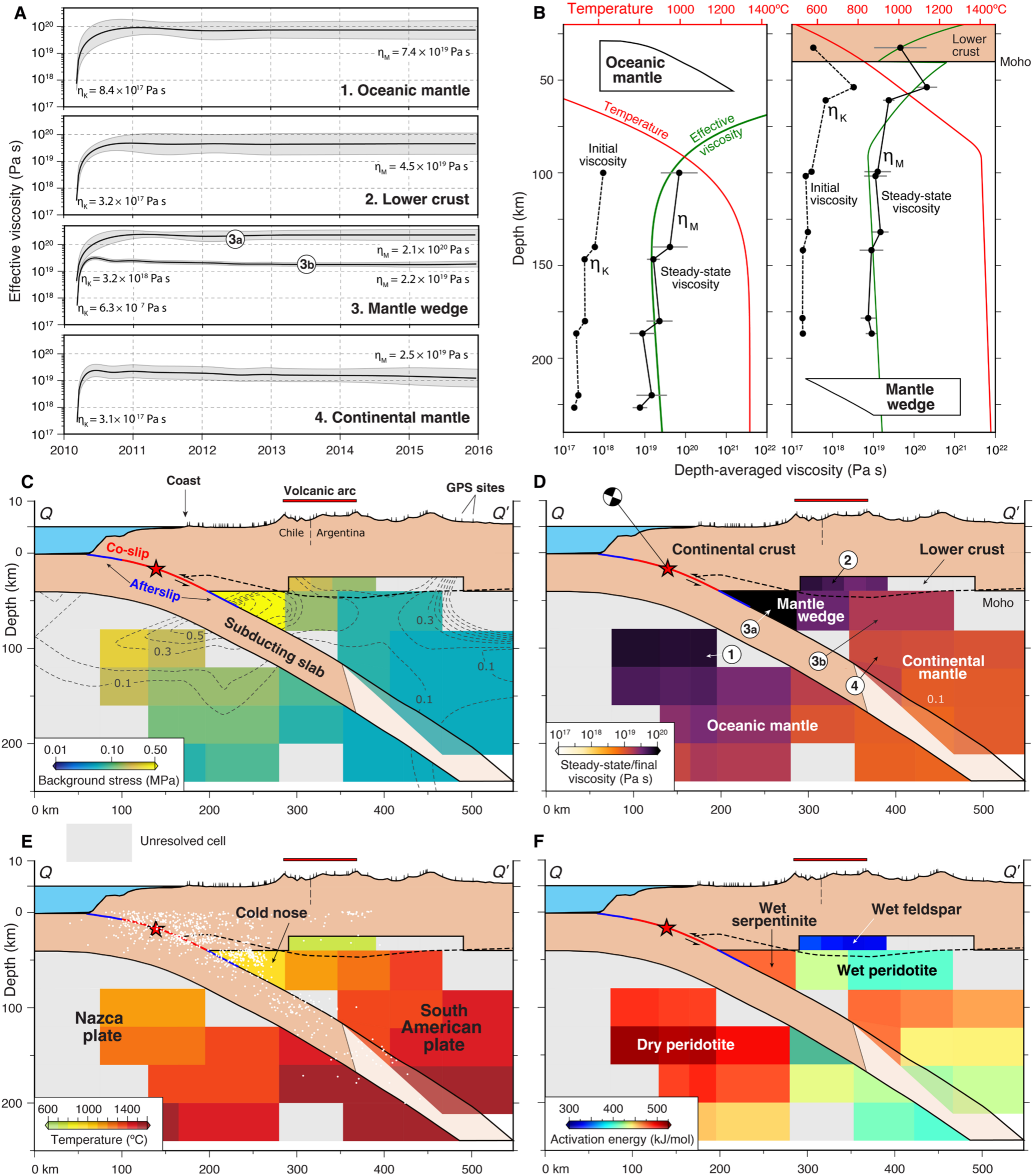
Rheological and thermal properties for the Maule region from the inversion and flow law modeling. (**A**) Time evolution of effective viscosity for a few well-resolved polyhedral volumes including two MW examples that highlight the high-viscosity MW corner (3a) compared with the adjacent sub/backarc upper mantle (3b). (**B**) Depth-averaged initial and steady-state viscosities for the oceanic and continental regions and best-fit theoretical temperature and viscosity profiles (Supplementary Text; table S1). (**C**) Background stress levels. Dashed gray contours show the resolution power for the ε_13_ strain component related to shear motion on the megathrust (fig. S6). Black dashed line represents the continental Moho. (**D**) Estimated steady-state viscosities η_M_. Circled numbers refer to the curves in (A). (**E**) Temperature estimates based on the rock properties constrained by the inverted steady-state stresses and strain rates assuming dislocation creep. White dots are 2000–2016 seismicity. (**F**) Activation energy (see the Supplementary Materials, tables S1 and S2, and fig. S8 for a full description of the associated calculations, best-fit rheological parameters, and additional derivatives including the initial viscosity η_K_, background strain rate, and temperature anomaly). The profile location is shown in Fig. 2.

We track the viscous strain rate and stress in the lithosphere-asthenosphere system to obtain an estimate of the temporal evolution of effective viscosity

$$\eta_{\text{eff}}(t)=\frac{\tau_{0}+\Delta \tau (t)}{{\dot{\epsilon }}_{0}+\Delta \dot{\epsilon }(t)}$$

for each polyhedral volume, where τ_0_ and ∆τ(*t*) are the background stress and postseismic stress change and ${\dot{\epsilon }}_{0}$ and $∆\dot{\epsilon }$ are the background and postseismic strain rate at each time step (Supplementary Materials; Fig. 5 and figs. S3 and S8) (*7*, *8*). The postseismic strain rate and associated stress are obtained directly from the Kalman filter of the geodetic data. We estimate the background strain rate and stress, assuming that the effective viscosity follows an exponential evolution (Supplementary Materials). The asymptotic value provides a proxy for the absolute stress, which decreases with depth as expected (Supplementary Materials; Fig. 5 and fig. S8). Other prominent features include a strong MW corner and a large viscosity contrast between the Nazca and South American plates. In a depth-averaged sense (Fig. 5B), the viscosity in the LC appears slightly higher than that in the CM and lower than that in the MW. We find that viscosities vary with time, with initial values of 10^17^ to 10^18^ Pa s, which rapidly increase during the first few weeks after the earthquake to steady-state values of 10^19^ to 10^20^ Pa s. This transient behavior lends support to the notion that a bi-viscous Burgers rheological model (*1*, *5*), with close to two orders of magnitude difference between initial and steady-state viscosities (*1*, *7*, *15*), is an adequate predictor of postseismic viscoelastic stress relaxation in the mantle beneath central Chile (Fig. 5 and figs. S3 and S8). Our estimated upper-mantle viscosities fall within the range of previous Maule postseismic studies (*23*, *27*).

## Rheological and thermal structure of the lower crust and upper mantle

**Fig. 6:**
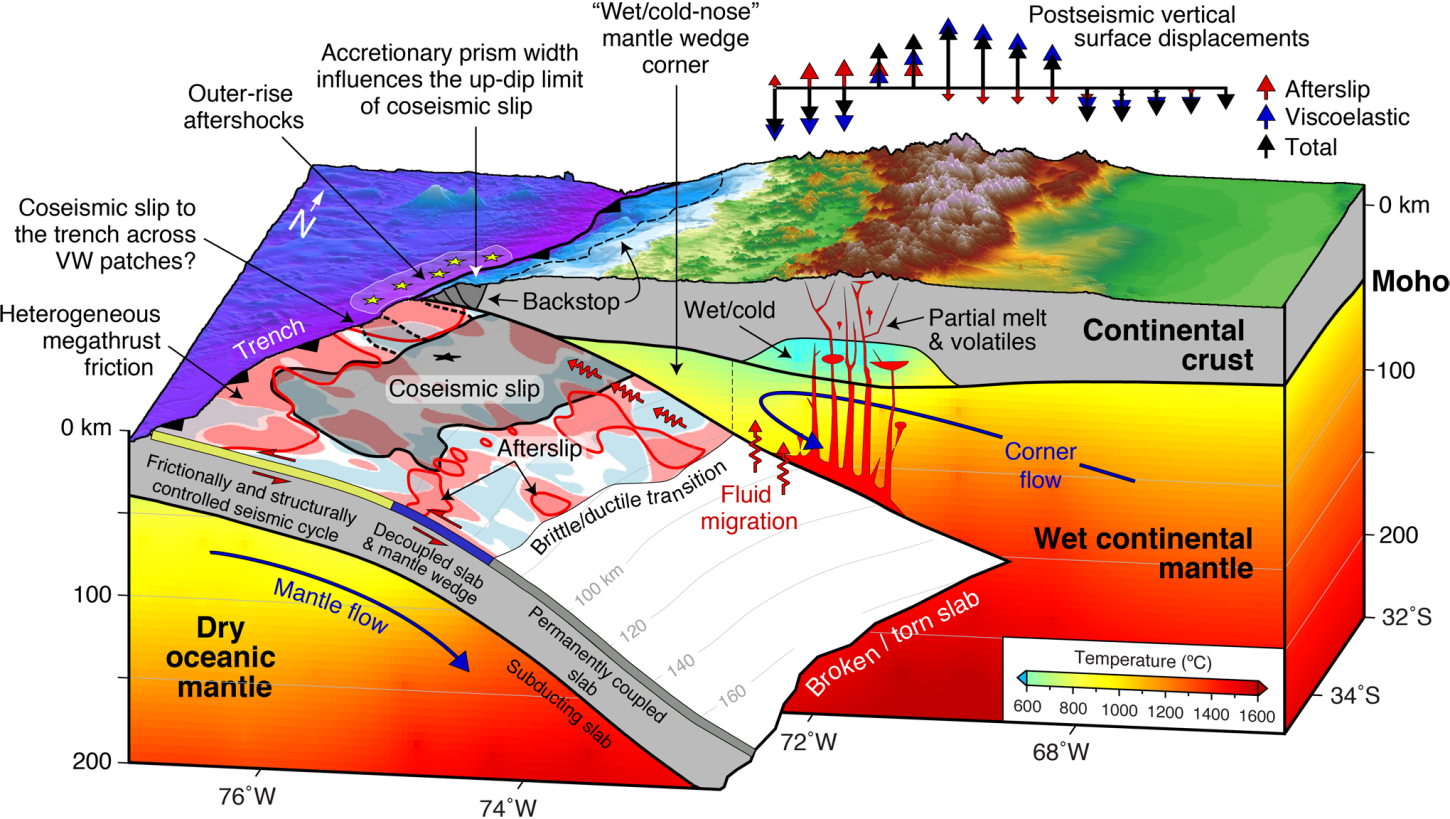
Conceptual view of the Maule subduction zone based on our results. The megathrust fault exhibits heterogeneous frictional properties characterized by a patchwork of regions with variable behavior (Fig. 4). The majority of coseismic slip occurred in a UW region, although our frictional properties are not well constrained across the seismogenic zone. The shallow megathrust beneath the narrow AP, where most of the afterslip occurred, is largely VS (Fig. 4). Significant slip may have reached the trench particularly adjacent to the zone of intense outer-rise aftershocks. Vertical slices show the interpolated steady-state temperature for the lithosphere-asthenosphere system. Key features include a “wet/cold-nose” MW corner and the adjacent wet/warm upper mantle and inferred zone of partial melt beneath the volcanic arc at the base of the wet/cold LC [see Fig. 5 for rheological interpretations and fig. S8 for a comparable cross-sectional view and the associated temperature anomaly (i.e., difference from depth-averaged mean), which further highlights prominent features in our thermal model]. Vertical arrows above the topography illustrate the schematic, first-order pattern of total postseismic vertical displacements including the afterslip and viscoelastic contributions. Other labeled features (e.g., coupled/decoupled zones) are discussed in the main text.

The length of the geodetic time series allows us to clearly identify the steady-state viscosity structure of the Central Chile subduction zone. In general, we find that stratified and spatially varying viscosities characterize the region surrounding the earthquake (Fig. 5 and fig. S8). In the ductile regions, rock strength is controlled by several parameters of which temperature is paramount (*40*). Assuming the dominance of dislocation creep with the constitutive relationship [i.e., flow law; (*3*, *41*)]

$$\dot{\epsilon }=A\tau^{n}{\left(C_{\text{OH}}\right)}^{r}e^{-\frac{Q+\mathrm{pV}}{\mathrm{RT}}}$$

where $\dot{\epsilon }$ is the strain rate, *A* is the pre-exponential factor, τ is the deviatoric stress, *n* is the stress exponent, *C*_OH_ and *r* are the water fugacity and associated exponent, respectively, *Q* is the activation energy, *p* is the confining pressure, *V* is the activation volume, *R* is the universal gas constant, and *T* is the temperature, we exploit the Arrhenius relationship between viscosity and temperature to interpret the rheological structure. We first assume constitutive parameter values within the uncertainties of experimental results for damp conditions (*41*) and solve for the temperature distribution in the lithosphere-asthenosphere system (Figs. 5 and 6, fig. S8, and tables S1 and S2). We then take an alternative approach and invert for the activation energy, which trades off with temperature, thus providing end-member, complimentary views of the rheology of deep crustal and upper mantle rocks.

As a preliminary step, we create 1D thermal profiles using a cooling half-space model for the oceanic lithosphere and the steady-state, heat conduction equation with elevated radiogenic heat production in the thick continental crust transitioning to an adiabatic gradient in the mantle (Supplementary Materials; Fig. 5). We compute the corresponding effective viscosities using the steady-state flow law and a grid search to estimate the best-fit rheological parameters (table S1). The results from this exercise closely agree with the depth-averaged viscosity profiles from our inversion, confirm the dominance of dislocation creep during the steady-state phase, and lend confidence that we can use the flow law, associated best-fit terms from the profiles, and the inverted, steady-state strain rate and stress to solve for the temperature of each resolved polyhedral volume (Figs. 5 and 6, fig. S8, and table S2). This provides us with a direct, independent estimate of temperature at these depths, complementing others based on numerical simulations (*40*). Temperature information is critical as it exerts a primary control on long-term subduction zone dynamics, arc volcanism, and the location and width of the seismogenic zone.

We then use the temperature estimates from the 1D profiles, the inverted strain rate and stress over the steady-state phase, and the dislocation creep flow law to solve for the activation energy *Q* associated with each of the volumes (Supplementary Materials; Fig. 5). To a first order, the main cause for variable *Q* is water content (*41*), and a comparison of our results with experimental estimates of *Q* (*3*, *16*) suggests that the OM is relatively dry, whereas the MW corner, the MW beneath the volcanic arc, and the LC are wet (Fig. 5).

The overall Maule viscosity and thermal structure is consistent with subduction zone models featuring sharp lateral contrasts between a “cold nose” (i.e., strong) forearc MW, a hot (i.e., weak) LC below the volcanic arc, and a warm backarc (Figs. 5 and 6, and fig. S8) (*6*, *27*, *40*, *42*). Our results are also consistent with stress-driven forward models (Supplementary Materials; fig. S9) and seismic tomography studies (*21*, *43*), which tend to lack either the resolution or coverage at comparable depths to permit a direct comparison with our upper mantle viscosity and thermal inversion results. We observe vertical stratification with a strong MW sandwiched between a weak LC and CM. The weak LC is wet, indicating the presence of partial melt beneath the volcanic arc, an elevated geothermal gradient, fluids, or a combination of these factors (*8*, *27*). Fluids released from the subducting slab migrate upward toward mid-crustal magma reservoirs beneath the active volcanoes, partially melting the LC to produce andesitic rocks (Fig. 6) (*44*). This mechanism has been proposed for subduction-related volcanic systems including the central Andes (*45*).

The strong, cold, and wet MW corner points toward the presence of fluid- and antigorite-enriched, semibrittle serpentinite (*43*), and the sharp thermal gradient imaged between the cold forearc and the hot and wet peridotic upper mantle beneath the volcanic arc (Fig. 5 and fig. S8) is also supported by surface heat flow measurements (*40*). The strength of the MW is controlled mainly by the thermal state and dehydration of the subducting slab, indicating a likely maximum depth of decoupling between the slab and overlying MW of ~70 to 80 km (*40*). The strengthened forearc MW can facilitate slow-slip events and nonvolcanic tremor (*22*, *30*) as fluids released from the slab across the decoupled zone stay in the subduction channel, form hydrated minerals, and participate in dehydration embrittlement reactions that create seismogenic conditions (Fig. 6). The spatial correlation between the wedge corner and seismicity supports this interpretation (Fig. 5). We attribute OM and CM viscosities that decrease with depth to the subduction of young (i.e., warm) oceanic crust and the associated release of fluids (*16*). Landward of the zone of decoupling, the sharp increase in the upper mantle temperature beneath the volcanic arc (fig. S8) implies that the slab and overriding mantle are strongly coupled, inducing MW flow and a pressure gradient that drives fluids countercurrent, bringing heat from depth and the backarc, resulting in high surface heat flow and arc volcanism (*40*, *46*).

The contrast in steady-state viscosity across the subducting slab at depths <150 km and the relatively high OM viscosity, in particular, is consistent with estimates derived from postseismic observations following the 1960 Chile, 2011 Tohoku-Oki, and 2012 Indian Ocean earthquakes (*14*–*16*, *18*). The CM viscosity reduction is attributed to subduction-related release of volatile-rich fluids, which serves to reduce the viscosity of the overlying CM and increase the MW water content (i.e., lower *Q*; Fig. 5) (*1*, *18*). At a depth of ~150 km, the temperatures of both the OM and CM are comparable (Figs. 5 and 6), supporting the suggestion that the slab dip increases moving southward across the region of coseismic slip and is detached or torn approaching the Mocha Fracture Zone (Figs. 1, 5, and 6) (*47*).

The dense regional geodetic network across the Central Chile subduction zone and associated long time series of surface deformation allow us to explore how afterslip and viscoelastic relaxation deform the lithosphere-asthenosphere system following a giant earthquake. These data, when combined with an innovative inversion approach, reveal lateral variations in frictional properties and a first-order thermal structure that controls the strength of the oceanic and continental lithosphere-asthenosphere system, furthering the notion that geodetic imaging is an important tool for determining the mechanical properties of subduction zones and better understanding the development of seismic hazard, complementing more traditional techniques such as seismic tomography.

## Supplementary Material

http://advances.sciencemag.org/cgi/content/full/5/12/eaax6720/DC1

Download PDF

Illuminating subduction zone rheological properties in the wake of a giant earthquake

## SUPPLEMENTARY MATERIALS

Supplementary material for this article is available at http://advances.sciencemag.org/cgi/content/full/5/12/eaax6720/DC1 Supplementary Materials and Methods Fig. S1. Continuous GPS network and interpolated interseismic velocity. Fig. S2. CGPS time series, empirical fits, and afterslip and viscoelastic flow modeling–based time series (also see subsequent pages). Fig. S3. Inversion parameters and sensitivity tests for select volumes. Fig. S4. Five checkerboard tests for slip on the megathrust and strain in the polyhedral volumes, which demonstrate our ability to resolve any up- and down-dip slip and viscous strain in the ductile region using our CGPS site distribution (white triangles). Fig. S5. Sensitivity tests for refining the model geometry. Fig. S6. Resolution power of strain components for each deformable finite volume. Fig. S7. Afterslip-related test and parameters for fault friction estimates. Fig. S8. Cross sections of derived rheological and thermal parameters from inversion and flow law modeling. Fig. S9. 3D, stress-driven, postseismic forward models of frictional afterslip and viscoelstic flow for comparison with inversion results. Table S1. Dislocation creep rheological parameter estimates for the Maule region. Table S2. Temperature estimates for the polyhedra. References (*48*–*67*)
