# Supplementary material for: Illuminating subduction zone rheological properties in the wake of a giant earthquake
Source: Sci Adv. 2019 Dec 18;5(12):eaax6720. doi: 10.1126/sciadv.aax6720 (PMC6989339; doi:10.1126/sciadv.aax6720)
Supplement: http://advances.sciencemag.org/cgi/content/full/5/12/eaax6720/DC1 [file supp_5_12_eaax6720__index.html]

Science Advances | Science AdvancesAAASSearchScience AdvancesMenu

## Supplementary Materials

**This PDF file includes:**

- Supplementary Materials and Methods
- Fig. S1. Continuous GPS network and interpolated interseismic velocity.
- Fig. S2. CGPS time series, empirical fits, and afterslip and viscoelastic flow modeling–based time series (also see subsequent pages).
- Fig. S3. Inversion parameters and sensitivity tests for select volumes.
- Fig. S4. Five checkerboard tests for slip on the megathrust and strain in the polyhedral volumes, which demonstrate our ability to resolve any up- and down-dip slip and viscous strain in the ductile region using our CGPS site distribution (white triangles).
- Fig. S5. Sensitivity tests for refining the model geometry.
- Fig. S6. Resolution power of strain components for each deformable finite volume.
- Fig. S7. Afterslip-related test and parameters for fault friction estimates.
- Fig. S8. Cross sections of derived rheological and thermal parameters from inversion and flow law modeling.
- Fig. S9. 3D, stress-driven, postseismic forward models of frictional afterslip and viscoelstic flow for comparison with inversion results.
- Table S1. Dislocation creep rheological parameter estimates for the Maule region.
- Table S2. Temperature estimates for the polyhedra.
- References (*48*–*67*)

Download PDF

**Files in this Data Supplement:**

- Adobe PDF - aax6720\_SM.pdf
