# Supplementary material for: Illuminating subduction zone rheological properties in the wake of a giant earthquake
Source: Sci Adv. 2019 Dec 18;5(12):eaax6720. doi: 10.1126/sciadv.aax6720 (PMC6989339; doi:10.1126/sciadv.aax6720)
Supplement: Download PDF [file aax6720_SM.pdf]

Science Advances
AAAS

advances.sciencemag.org/cgi/content/full/5/12/eaax6720/DC1

Supplementary Materials for

Illuminating subduction zone rheological properties in the wake of a giant earthquake

Jonathan R. Weiss, Qiang Qiu*, Sylvain Barbot, Tim J. Wright, James H. Foster, Alexander Saunders, Benjamin A. Brooks, Michael Bevis, Eric Kendrick, Todd L. Ericksen, Jonathan Avery, Robert Smalley Jr., Sergio R. Cimbaro, Luis E. Lenzano, Jorge Barón, Juan Carlos Báez, Arturo Echalar

*Corresponding author. Email: qiuqiang@ntu.edu.sg

Published 18 December 2019, *Sci. Adv.* **5**, eaax6720 (2019)
DOI: 10.1126/sciadv.aax6720

This PDF file includes:

Supplementary Materials and Methods
Fig. S1. Continuous GPS network and interpolated interseismic velocity.
Fig. S2. CGPS time series, empirical fits, and afterslip and viscoelastic flow modeling–based time series (also see subsequent pages).
Fig. S3. Inversion parameters and sensitivity tests for select volumes.
Fig. S4. Five checkerboard tests for slip on the megathrust and strain in the polyhedral volumes, which demonstrate our ability to resolve any up- and down-dip slip and viscous strain in the ductile region using our CGPS site distribution (white triangles).
Fig. S5. Sensitivity tests for refining the model geometry.
Fig. S6. Resolution power of strain components for each deformable finite volume.
Fig. S7. Afterslip-related test and parameters for fault friction estimates.
Fig. S8. Cross sections of derived rheological and thermal parameters from inversion and flow law modeling.
Fig. S9. 3D, stress-driven, postseismic forward models of frictional afterslip and viscoelstic flow for comparison with inversion results.
Table S1. Dislocation creep rheological parameter estimates for the Maule region.
Table S2. Temperature estimates for the polyhedra.
References (*48*–*67*)

Supplementary Materials and Methods

GPS data processing and preparation

For the Maule postseismic analysis we include continuous GPS (CGPS) data from a large regional geodetic network (fig. S1). For each daily solution we process all available data using GAMIT (*48*), which estimates the 3-D relative position of ground stations and satellite orbits and GLOBK (*49*), which merges the daily regional with global solutions for International Global Navigation Satellite Systems Service (IGS) sites. Since we are interested primarily in regional deformation, we refrain from the global stacking common to large-scale scale plate motion studies and instead stack only stations on the South America and Nazca Plates and a few sites on adjacent plates and minimize motions of stations located on the stable core of the South American craton. We express our daily time series in a craton-fixed reference frame with horizontal and vertical velocities of ~1mm/yr. Specifically, we use 44 CGPS stations primarily located in Brazil to create the horizontal reference frame. These sites have a total RMS horizontal velocity of 0.75 mm/yr. The total RMS velocity of the 35 sites used to constrain the vertical reference frame is 1.13 mm/yr. Our GPS field, processing, and time series analysis methods in South America are described in detail elsewhere (*50*).

Prior to modeling, we isolate the postseismic portion of the CGPS time series by removing an estimate of the interseismic velocity. For sites where sufficient data exist prior to the Maule earthquake (~2 years) we do this by simultaneously estimating the best-fitting interseismic velocity, coseismic step, postseismic decay function, and seasonal component in a least squares sense (*50*). We subtract the interseismic and coseismic contributions to the time series but not the

estimated seasonal term as our tests suggest that subtracting this component often results in an increase in the time series scatter.

For CGPS sites with little or no pre-Maule data, we require an estimate of the associated interseismic velocity, which we derive by combining the best-fit North, East, and Up velocities from sites with pre-quake data (described above) with published interseismic velocity estimates for the Maule region (fig. S2) (*24, 51-55*). We use the most recent velocities for sites with numerous solutions and only include those expressed in a South America-fixed reference frame, which show relative uniformity and no noticeable reference frame bias. We calculate a spatially averaged dataset using the velocity uncertainties as weighting factors, create interpolated North, East, and Up velocity fields (fig. S2), extract velocities for locations with little to no pre-quake data, and remove these estimates prior to computing the best-fit postseismic decay functions.

For daily position uncertainties we calculate the mean and standard deviation of the time series in a 7-day moving window, multiply the standard deviation by 1.5, and assign this value as the uncertainty for daily positions within the corresponding time window. We fill data gaps using the aforementioned best-fit postseismic decay functions and assign uncertainties to these daily positions by taking the mean of the weekly standard deviation estimates and multiplying this value by 10 so that the empirical fits across data gaps are down-weighted during the afterslip and viscoelastic strain inversion.

Modeling approach

We use SLAB1.0 (*56*) to define the geometry of the subduction zone interface, which we discretize into a network of triangular boundary elements that extend along strike for ~400 km and from the trench to a depth of ~75 km (main text Fig. 3). We simultaneously invert the postseismic surface displacement time series at each CGPS site for localized afterslip on the megathrust and distributed deformation in the ductile regions using a modified version of the Kalman-based extended network inversion filter (*7*, *57*, *58*), which provides conditional probability estimates for the model parameters based on uncertainty in the data. The model parameters in this case correspond to the afterslip magnitude and direction for each of the triangular boundary elements (*59*) and six independent components of strain for each of the polyhedral volume elements (*28*) and their associated rates. The volumes vary in size, but their bases are typically 50-100 km wide/long and their heights range from 10-100 km. A linear summation of the six strain components determines the total deformation of each volume element and the 3D Green's functions map the strain at depth to the surface displacements. The amplitudes of the strain and fault slip Green's functions determine our ability to successfully explain the associated surface displacements and reveal tradeoffs between the two different deformation mechanisms. Due to the large number of model parameters we make simplifying assumptions to make the inversion more tractable and to force smooth parameter distributions. These include a no back-slip, or positivity penalization, during the afterslip inversion and we assume that the viscoelastic flow is deviatoric, penalizing strain directions perpendicular to the induced coseismic stresses and promoting strain directions that align with the coseismic stress directions. We also penalize afterslip so that it preferentially occurs in the region surrounding the coseismic rupture patches and we apply spatial smoothing using stress kernels to both the afterslip and strain components to avoid large variations in slip or strain between neighboring

elements that over-fit the postseismic time series. The temporal and spatial smoothing hyper-parameters are estimated simultaneously with the model parameters (*7*, *57*, *58*). Following the previous work (*7*), these smoothing hyper-parameters are determined iteratively until the best fit to the GPS measurements is achieved.

It is impossible to directly measure the background stress and strain rate of the upper mantle. Therefore, we again follow previous work (*7*) when estimating the total stress for evaluating the time evolution of effective viscosity for each deformable volume and consider three stress components; the initial stress from the coseismic rupture, the stress evolution for each strain volume, and the background stress corresponding to the steady-state viscosity multiplied by the background strain rate. We perform a grid search for the background strain rate and steady-state viscosity that best explain the time evolution of the effective viscosity curves compared to the Burgers rheology model (Figs. 5 and S6; *7*). The minimum misfit provides the most likely combination of both background viscosity and strain rate that matches the trend of stress relaxation within the polyhedral volumes and the corresponding effective viscosity time series.

Fault friction

To explore the frictional properties of the megathrust we assume that the temporal evolution of afterslip obeys the rate-and-state friction law under steady-state, with

$$\tau_{\mathrm{ss}} = \mu^{*}\sigma_{\mathrm{n}} + (a-b)\sigma_{\mathrm{n}} log \frac{V}{V^{*}}$$

where a and b are the rate-and-state frictional parameters (*a-b* describes the fault behavior), τ_{ss} the driving shear stress, σ_n the effective normal stress, μ^* the reference coefficient of friction, and V^* and V the reference slip velocity and slip velocity, respectively. We estimate *(a-b)* σ_n (equivalent to the ratio of Coulomb shear stress change to slip rate) for each fault patch

$$(a-b)\sigma_{\mathrm{n}} = \frac{d\Delta CFS}{\mathrm{dlog}(V)}$$

where *ΔCFS* is the time-varying Coulomb stress on the creeping fault element (*4, 10, 31, 60*). If *a-b* > 0 the material is velocity strengthening and primarily stable sliding (i.e., creep) is possible. In contrast, if *a-b* < 0, the material is velocity weakening and potentially seismic rupture (i.e. frictional instabilities may nucleate).

Steady-state rheology, temperature, and activation energy estimates

The rheological properties (i.e. constitutive stress-strain rate relationship) of olivine aggregates in the mantle determined from deformation experiments are described using a flow law in the form

$$\dot{\varepsilon} = A\sigma^n d^{-m}(C_{OH})^r exp\left(-\frac{Q+pV}{RT}\right)$$

where $\dot{\varepsilon}$ is the strain rate, A is a pre-exponential factor, σ is deviatoric stress, n is the stress exponent, Q is the activation energy, R is the universal gas constant, T is the temperature, V is the activation volume, p is the confining pressure, d is grain size, m is the grain size exponent,

and C_{OH} and r are the water concentration and associated exponent (*16, 41, 61*). Diffusion creep is associated with $n = 1$ and $m = 2 - 3$, and dislocation creep is associated with $n = 3 - 5$ and $m = 0$. To calculate the geothermal gradient of the continental lithosphere-asthenosphere system required for the depth-dependent viscosity we solve the steady-state, 1-D heat conduction equation with radiogenic heat production using Equation (3) of Afonso and Ranalli (*62*) with an adiabatic gradient of 0.3 °C/km (*63*) and values of heat production, thermal conductivity and density from Currie and Hyndman (*64*). For the oceanic mantle, a cooling half-space model was used to calculate the geothermal gradient assuming a 35 Ma subducting plate (*44*), considering the cooling effect of the 40-km-thick subducting slab. We compute the effective viscosity profile using the stress-strain rate relationship equation given above and estimate the best-fit terms via trial-and-error. The estimated parameters (table S1) are in agreement with those from laboratory experiments (*3*, *65*).

Based on these best-fit terms, we can further investigate the temperature and water content (i.e. activation energy) of the Maule lower crust and upper mantle with two end-member approaches. The first involves using the best-fit rock properties from the 1-D profiles (Fig. 5 and table S1) to estimate the 3-D temperature structure. We assume the rocks within the oceanic mantle, continental mantle and lower crust have the same properties (e.g. the mantle wedge has the same best-fit rock properties as the continental mantle). With this assumption and the inverted strain rate and stress over the steady-state stage (e.g., Figs. 5 and S5), we solve for the temperature of each resolved polyhedron using the stress-strain rate relationship equation shown above. The corresponding temperature values and their associated uncertainties are given in table S2. The second approach uses the 1-D temperature profiles for the oceanic and continental mantle and the

stress-strain rate relationship equation to solve for the activation energy Q of each resolved polyhedron (Fig. 5). These two approaches capture temperature and water content variations and together provide insight into the physical properties of rocks in the mantle in the Chile subduction zone. Both approaches are simplifications and represent end-member scenarios as in reality there will be mixed lateral variations of temperature and water content and therefore activation energy. See the main text for a more detailed description of the temperature and activation energy results.

3D stress-driven forward modeling with UniCyclE

We conducted a small suite of stress-driven forward models using the Unified Cycle of Earthquakes (UniCyclE) numerical code, which simulates fault slip and viscoelastic flow during the earthquake cycle (*9, 66*), to help evaluate first-order results of our inversion (fig. S9). We do not attempt to explain all of the details of the inversion, which intrinsically captures the spatial variability of afterslip and viscous strain without *a priori* rheological assumptions. Many initial assumptions are required to achieve satisfactory forward model fits to the data and large uncertainties exist in the stresses produced by coseismic slip models, which are also highly variable depending on the datasets and inversion technique used to produce them. To facilitate the comparison, we design a series of forward numerical experiments guided by our inversion results where we consider both bi-viscous, Burgers and nonlinear, power-law rheologies for the viscoelastic flow coupled with nonlinear transient creep for the afterslip (*16*).

For the Burgers model, we use the inverted initial and steady state viscosities (η_K and η_M) along profile Q-Q’ (Figs. 2 and 5). For simplicity, we expand these values by assuming there are no

along-strike variations in viscosity. For fault friction, we assume spatially uniform frictional coefficients but slightly reduce the friction on the shallow portion of the fault to force more afterslip in the up-dip direction. This is done to maintain consistency with the inversion results while staying within the range of the inverted values (Fig. 4).

The Burgers rheology forward model does a nice job of reproducing the horizontal displacements (fig. S9a) but does not do well with the vertical displacements (fig. S9b). For example, the model predicts subsidence across the volcanic arc whereas the GPS data reveal wholesale uplift. The vertical misfits are somewhat expected and may be due to initial condition uncertainties, coarse sampling of the strain volumes, or the assumed rheology since we know the vertical component is very sensitive to the specified rheology (*3*, *7*, *14*). Overall the inversion results provide a decent initial estimate for the stress-driven forward model.

We next employ a nonlinear, power-law rheology with transient creep. The rheological parameters are from the 1D fitting exercise described in the main text and Supplementary Materials (Fig. 5 and table S1) while the fault friction coefficients are the same as those described above. In general, this model overestimates the deformation field magnitude, with larger near- to mid-field displacements and significantly more strain in the mantle wedge compared to the Burgers model. The power-law model also does a poor job of reproducing the vertical displacements.

For our third forward calculation we recall that the inversion results suggest the mantle wedge is strong (i.e. relatively high viscosity) and thus is less deformed than the surrounding rocks during

the postseismic period. Therefore, we penalize strain in the mantle wedge volumes and find that the model does a much better job of fitting both the horizontal and vertical displacements compared to the previous forward models. The penalized model predicts coastal subsidence and uplift across the forearc but subsidence across the volcanic arc. All of these features are consistent with both the data and the best model from the inversion pointing towards a power-law rheology.

To summarize, our UniCyclE 3D stress-driven forward models confirm that the inversion results provide a good first order estimate of the subduction zone rheological structure. A nonlinear power-law rheology is preferred, and the strong mantle wedge corner is a robust feature from both the inversion and forward modeling exercises. We reserve additional forward modeling exercises for a follow-up study.

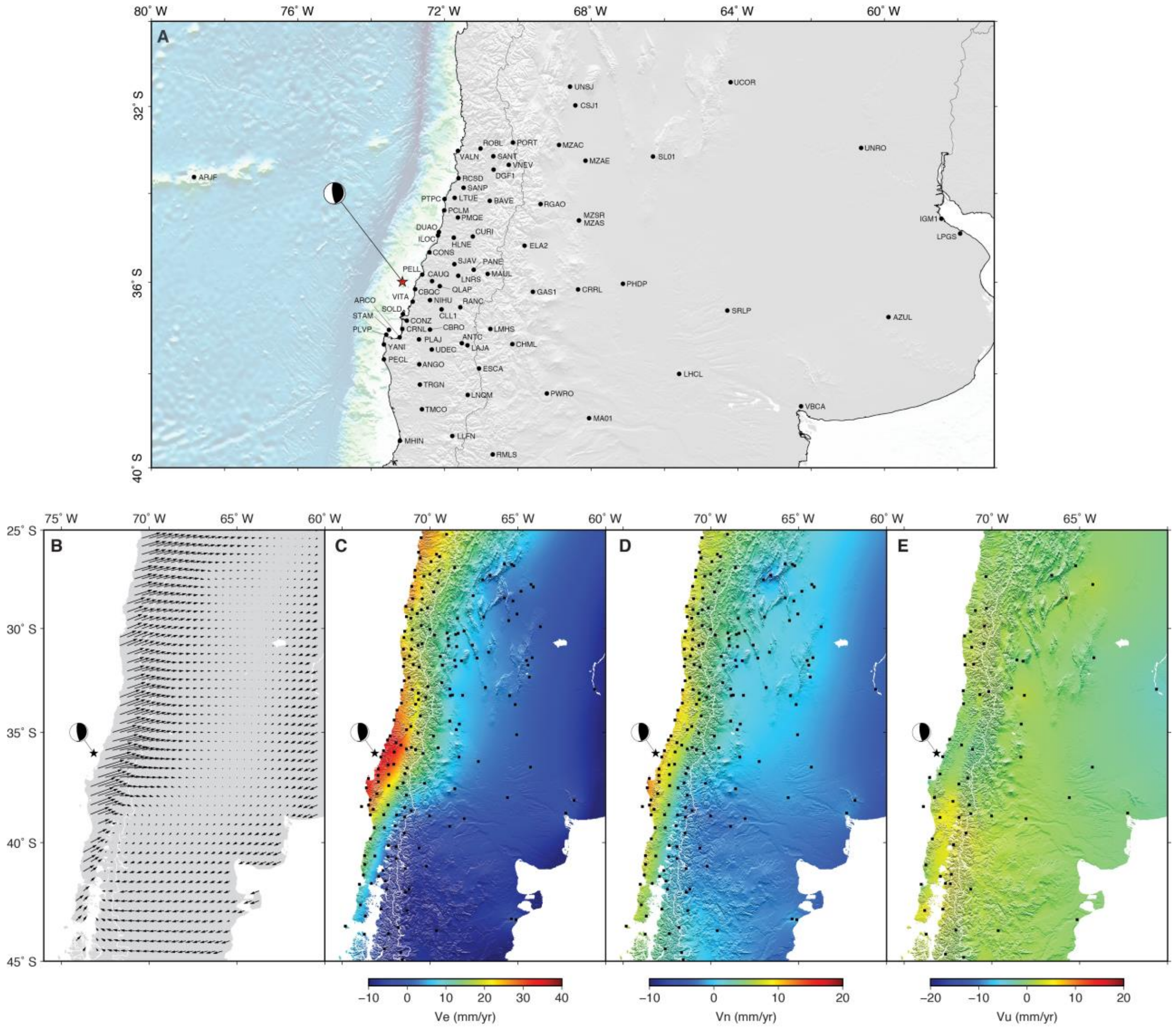

Fig. S1. Continuous GPS network and interpolated interseismic velocity. (**A**) Distribution of CGPS sites used in the analysis overlain on semi-transparent, hill-shaded SRTM topography (grays) and bathymetry (blues/greens). The 2010 M_w 8.8 Maule, Chile earthquake centroid location (red star) and focal mechanism are also shown. See fig. S2 for associated time series and model fits. (**B**-**E**) Interseismic velocity field used for sites with no pre-quake data. (B) Interpolated horizontal velocity field. (C-E) Interpolated North, East, and Up velocities. Black squares represent sites with published interseismic velocities used for the interpolation.

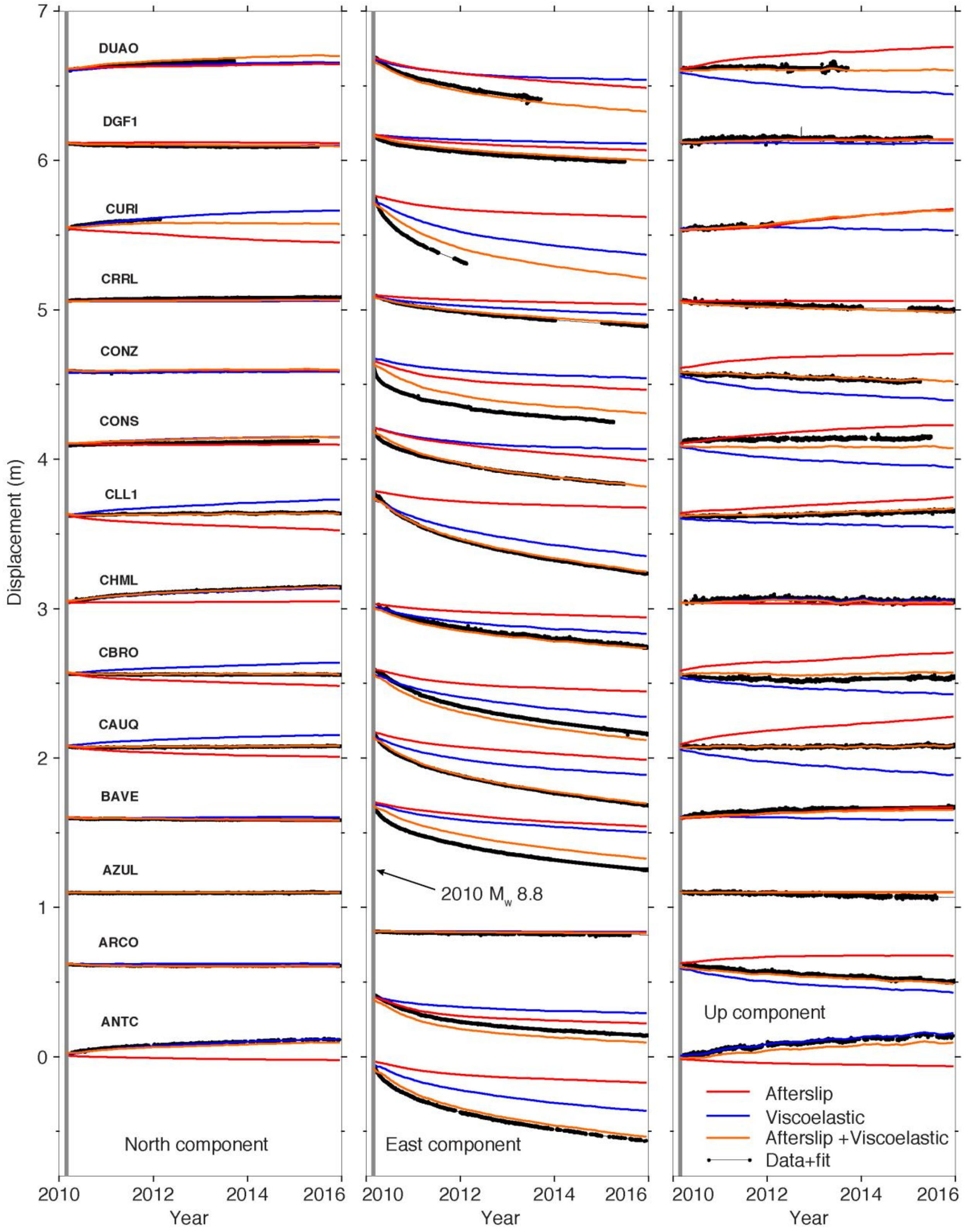

Fig. S2. CGPS time series, empirical fits, and afterslip and viscoelastic flow modeling–based time series (also see subsequent pages).

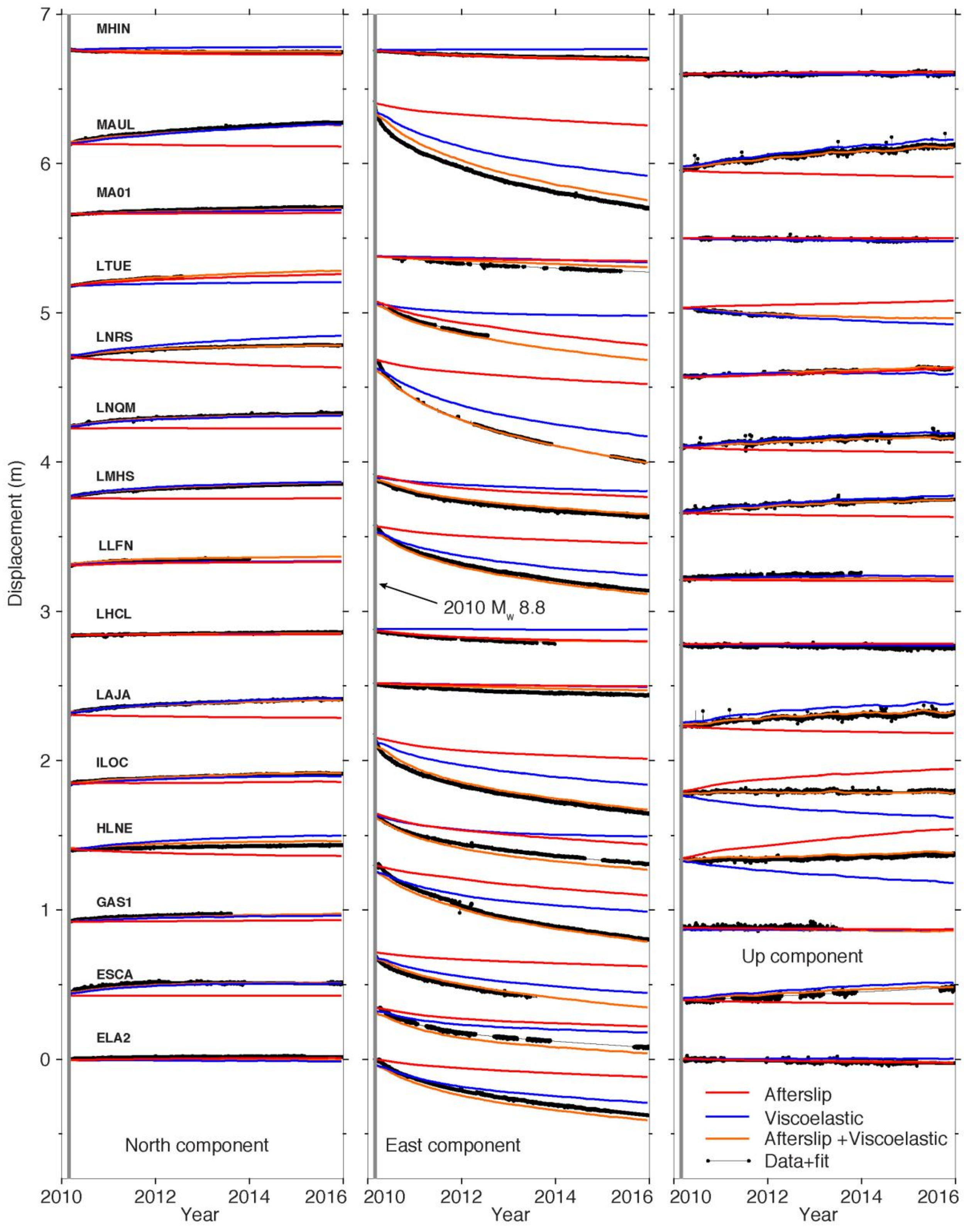

MHIN
MAUL
MA01
LTUE
LNRS
LNQM
LMHS
LLFN
LHCL
LAJA
ILOC
HLNE
GAS1
ESCA
ELA2
Displacement (m)
North component
East component
Up component
2010 M_w 8.8
Afterslip
Viscoelastic
Afterslip +Viscoelastic
Data+fit
Year

Fig. S2 cont.

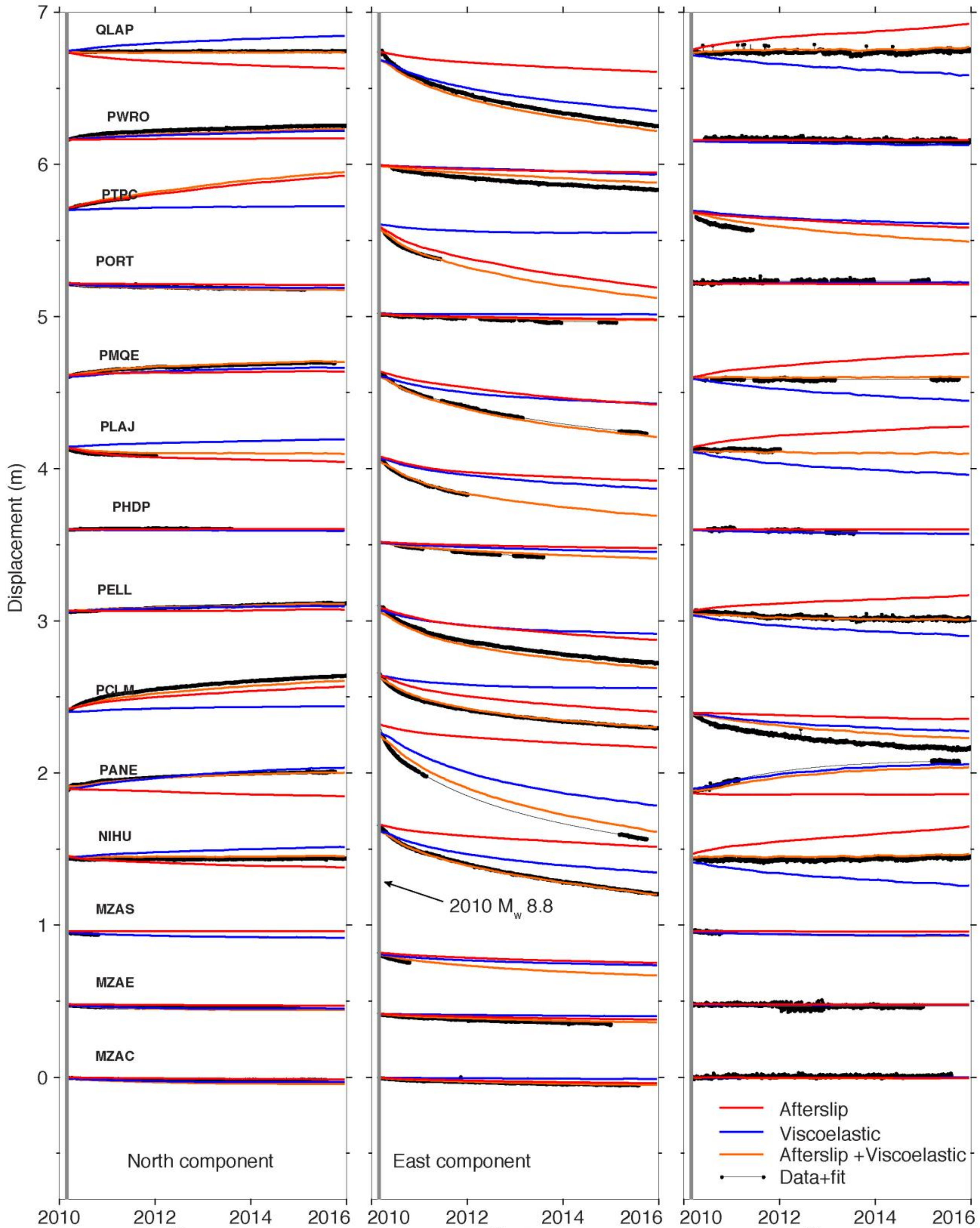
QLAP
PWRO
PTPC
PORT
PMQE
PLAJ
PHDP
PELL
PCLM
PANE
NIHU
MZAS
MZAE
MZAC
Displacement (m)
2010 M_w 8.8
North component
East component
Afterslip
Viscoelastic
Afterslip +Viscoelastic
Data+fit
Year
Year
Year

Fig. S2 cont.

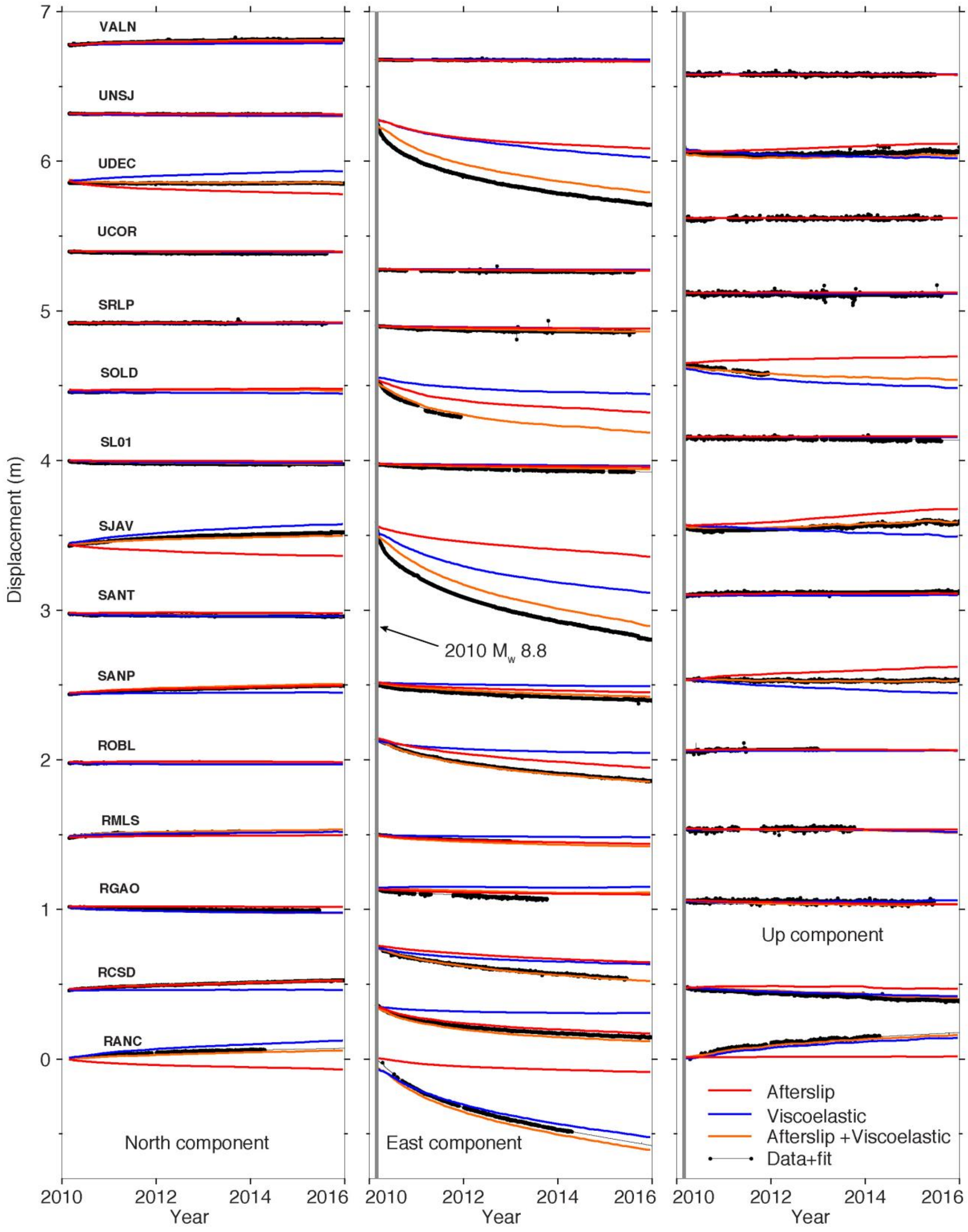

VALN
UNSJ
UDEC
UCOR
SRLP
SOLD
SL01
SJAV
SANT
SANP
ROBL
RMLS
RGAO
RCSD
RANC
Displacement (m)
North component
East component
Up component
2010 M$_w$ 8.8
Afterslip
Viscoelastic
Afterslip +Viscoelastic
Data+fit
Year

Fig. S2 cont.

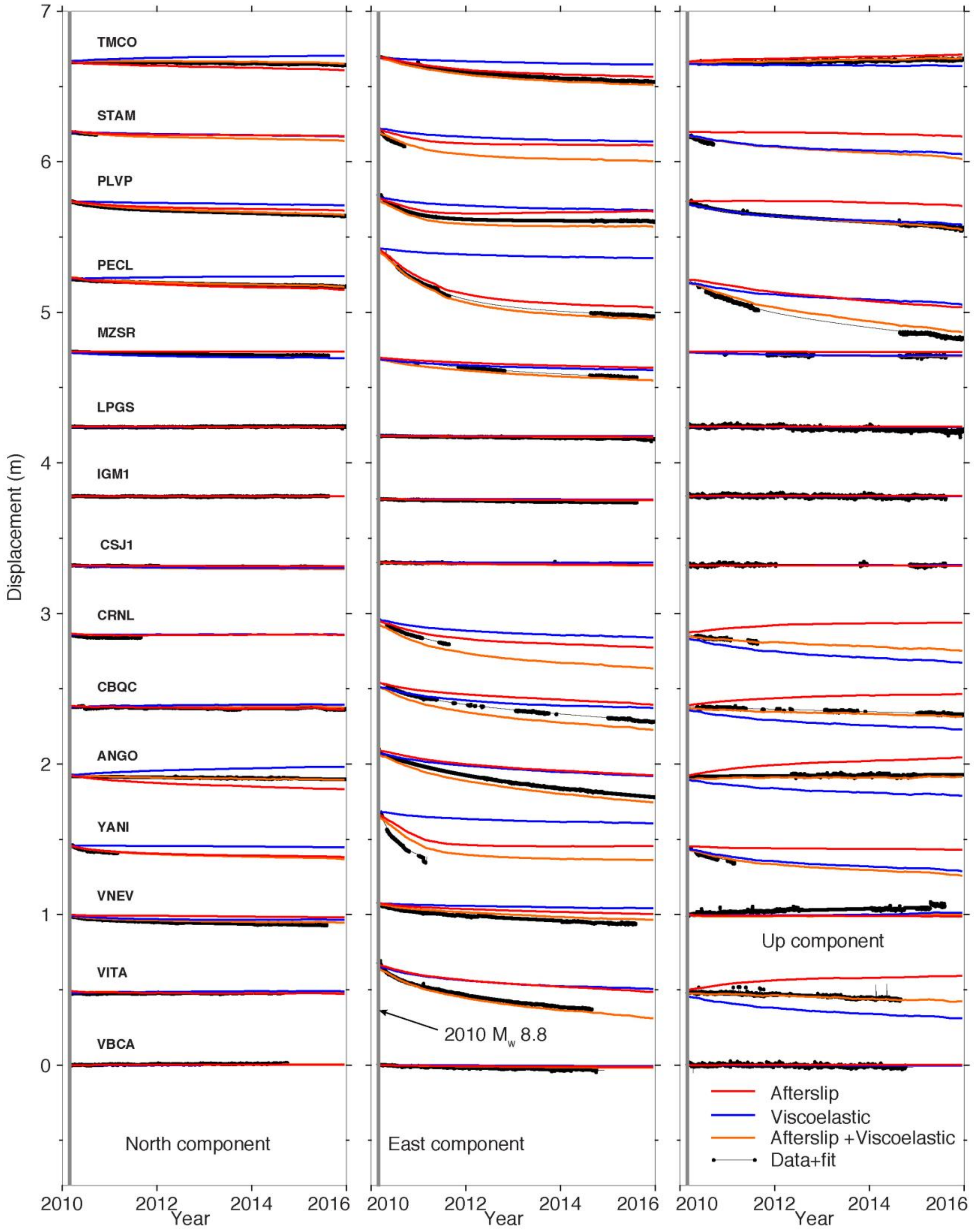

TMCO
STAM
PLVP
PECL
MZSR
LPGS
IGM1
CSJ1
CRNL
CBQC
ANGO
YANI
VNEV
VITA
VBCA
Displacement (m)
North component
East component
Up component
2010 M_w 8.8
Afterslip
Viscoelastic
Afterslip +Viscoelastic
Data+fit
Year

Fig. S2 cont.

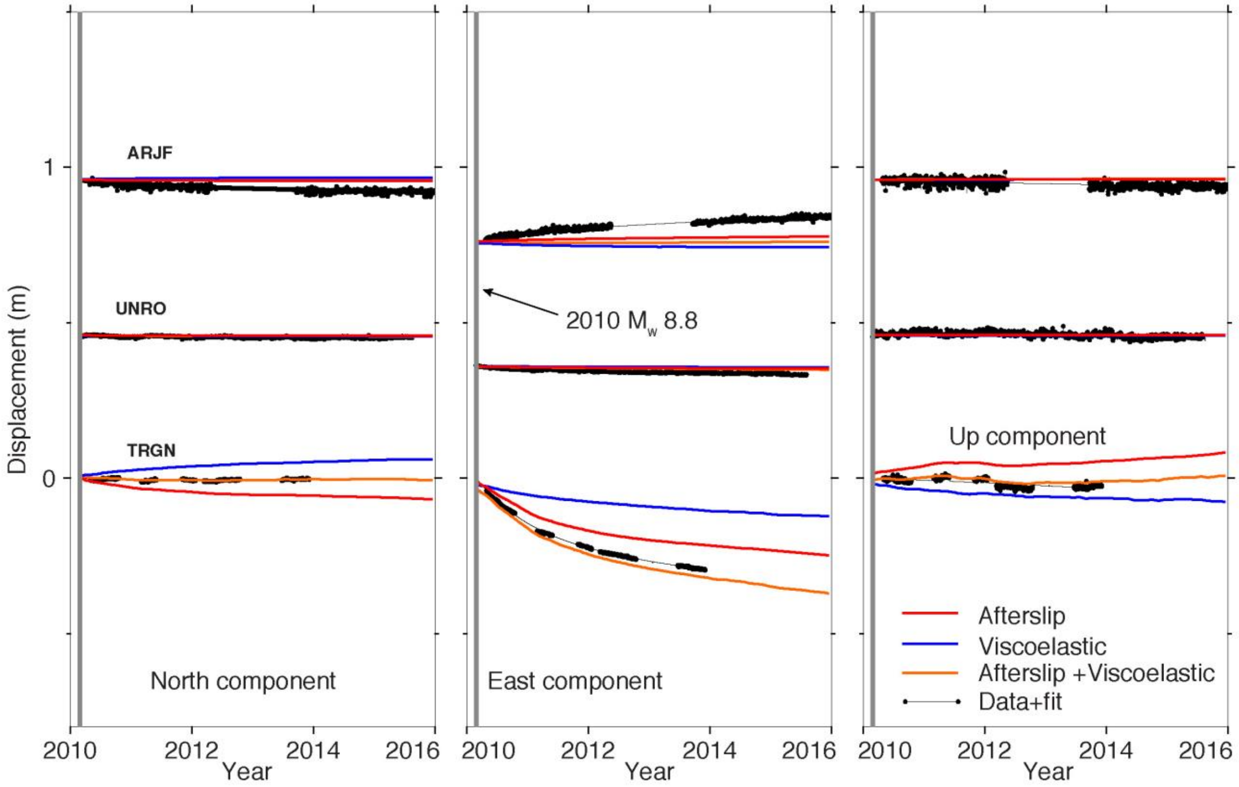

ARJF
UNRO
TRGN
Displacement (m)
1
0
2010 M_w 8.8
North component
East component
Up component
Afterslip
Viscoelastic
Afterslip +Viscoelastic
Data+fit
2010
2012
2014
2016
Year

Fig. S2 cont.

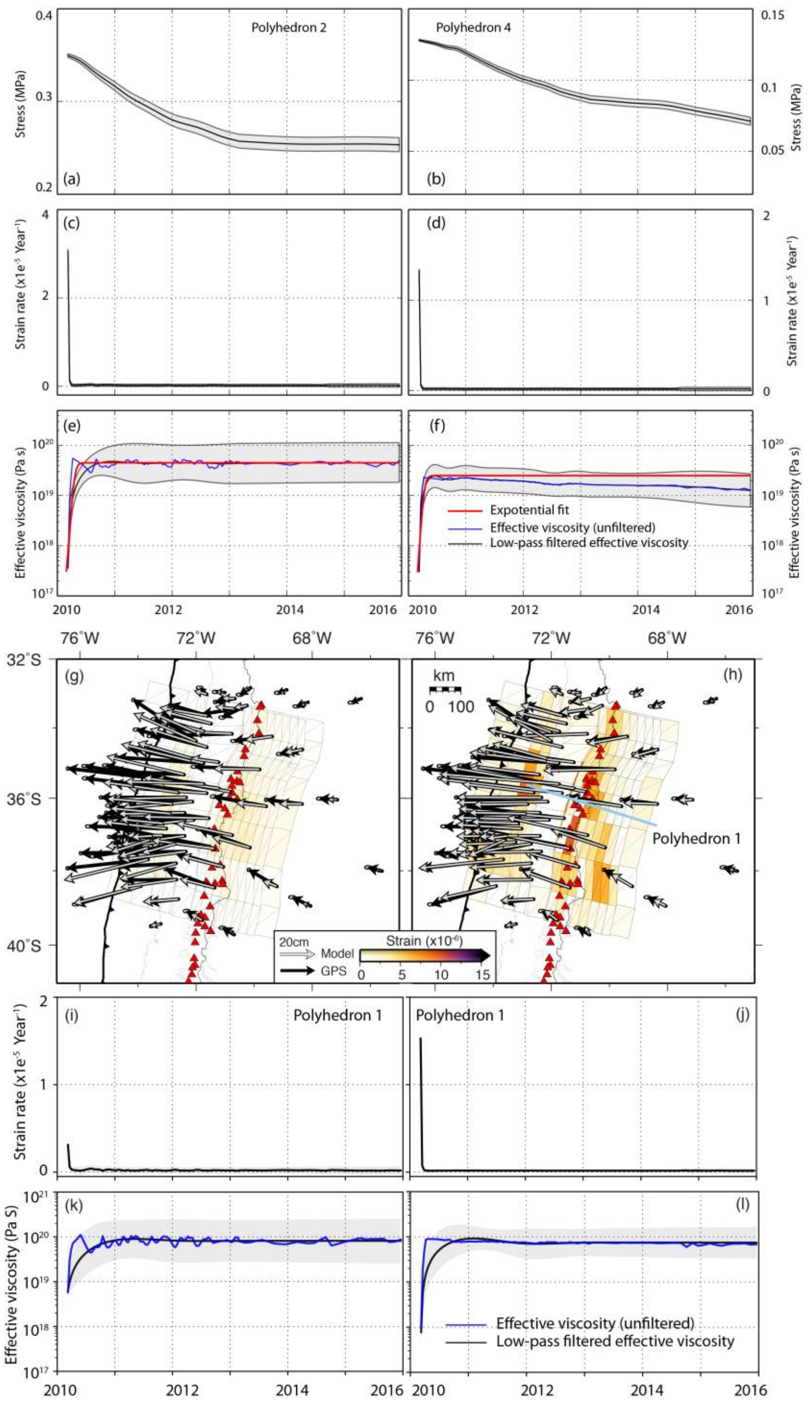

Fig. S3. Inversion parameters and sensitivity tests for select volumes. (**a-f**) Stress, strain rate, effective viscosity and functional fits for two example polyhedral volumes. Their locations are shown in Fig. 5 of the main text. (**g-l**) Sensitivity tests for the strain directivity constraints. (Left) Poor model results (loose constraints on the strain direction pointing to the coseismic stress). (Right) Final good model results (tight constraints). (g) Poor model-data fit for loose constraints. (h) Final model with good model-data fit for tight constraints. (i) Strain rate predicted from model (g) at polyhedron 1 labeled in (h). (i) Strain rate at polyhedron 1 for model (h). (k-l) The effective viscosity curves for the models in (g) and (h), respectively.

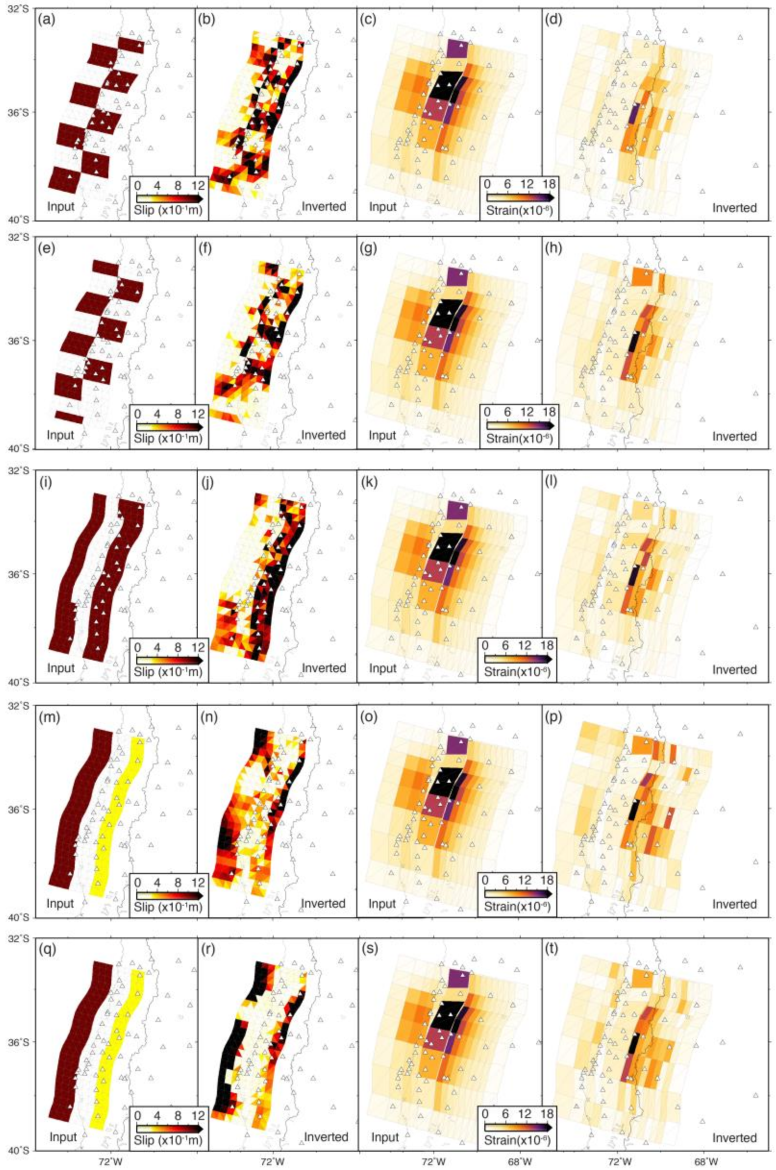

Fig. S4. Five checkerboard tests for slip on the megathrust and strain in the polyhedral volumes, which demonstrate our ability to resolve any up- and down-dip slip and viscous strain in the ductile region using our CGPS site distribution (white triangles). We use the same measurement covariances as with the actual postseismic time series and we use the amplitude of the coseismic stress change (second invariant of the stress tensor) to scale the viscous strain when creating the synthetic observations (*7*). We have explored the effect of spatial smoothing on the slip magnitude/pattern and our final results (Fig. 3) represent the model that best balances this effect for all the checkerboard tests. (**a-d**) Unit slip input with checkerboard size ~90 km along-strike and ~120km across strike, the corresponding recovered inverted slip (i.e. from the inversion), input viscous strain (the second invariant of the strain tensor), and recovered viscous strain. (**e-h**) Same as (a-d) with different slip input arrangement. (**i-l**) Checkerboard test with comparable slip on the shallow and deep portions of the fault. (**m-p**) Test with different amounts of slip on shallow and deep portions of the fault and no penalization in the region of coseismic rupture. (**q**-f) Same as (m-p) but the coseismic rupture region is penalized in the inversion. This test shows that the shallow and deep afterslip and the spatial pattern of strain are well resolved.

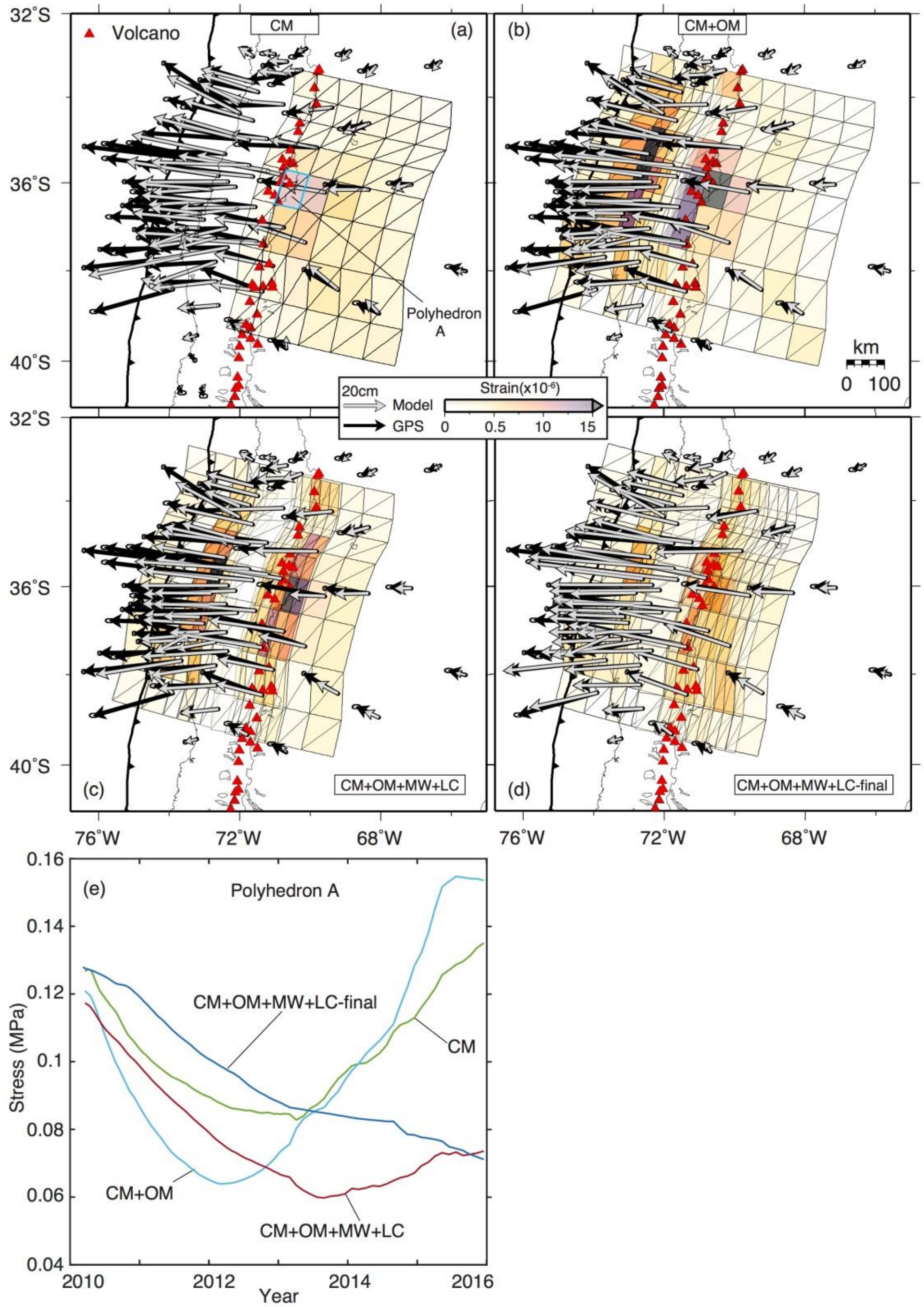

Fig. S5. Sensitivity tests for refining the model geometry. (**a**) Volumes in the continental mantle (CM) only. (**b**) Volumes in the continental and oceanic mantle (CM+OM). (**c**) Volumes in the continental mantle, oceanic mantle, mantle wedge and lower crust (CM+OM+MW+LC). (**d**) Volumes in the continental mantle, oceanic mantle, mantle wedge and a wider (to the east) lower crust (CM+OM+MW+LC-final). (**e**) Stress evolution curves for each of the model setups for one of the polyhedra labeled in (a). Only our final, combined geometry (CM+OM+MW+LC-final) shows stresses that continue to decay with time in the years following the mainshock. The afterslip contribution is included in each of the models.

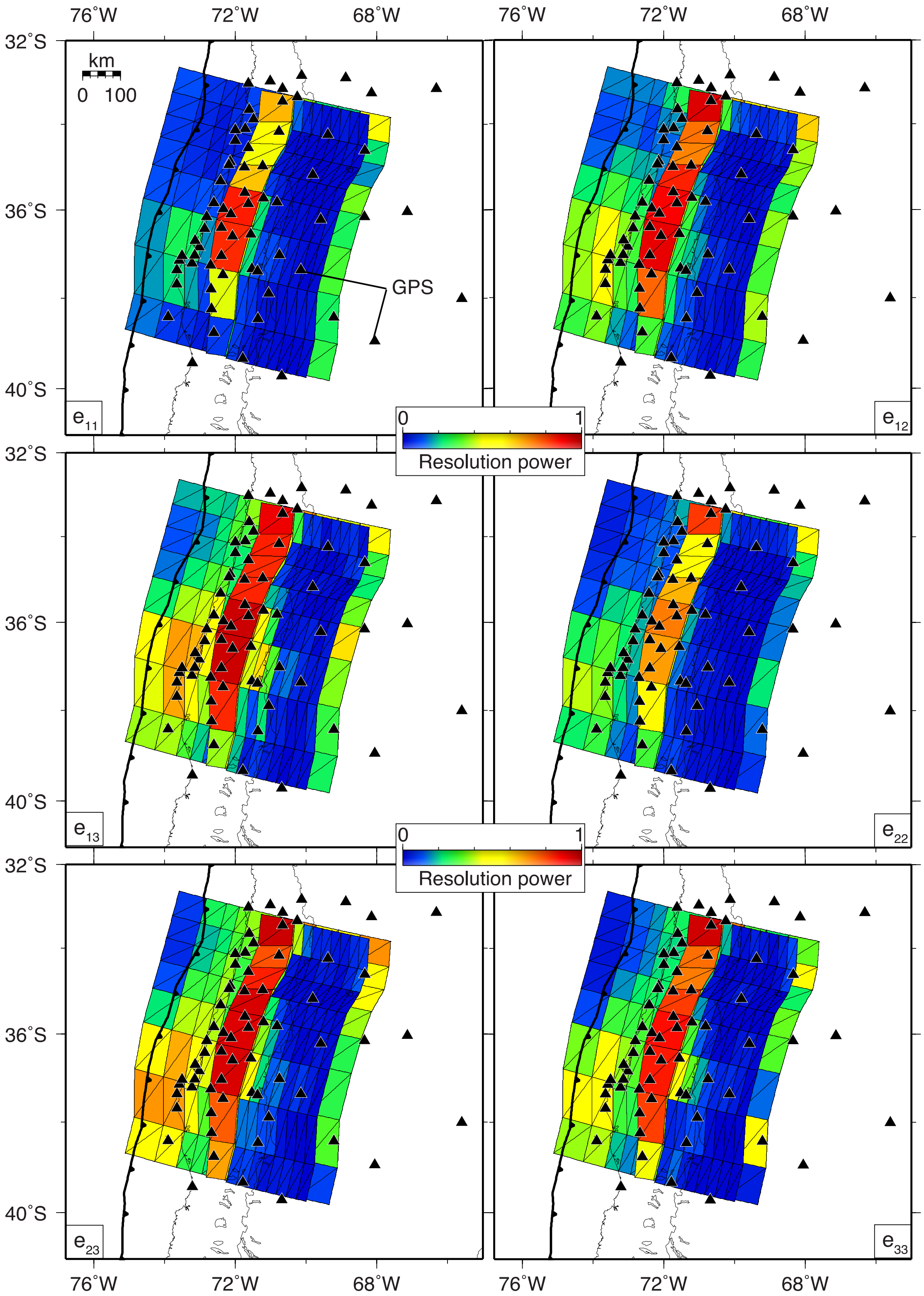

Fig. S6. Resolution power of strain components for each deformable finite volume. The color scale shows the resolution power with values equal to 0 (unresolved) and 1 (fully resolved).

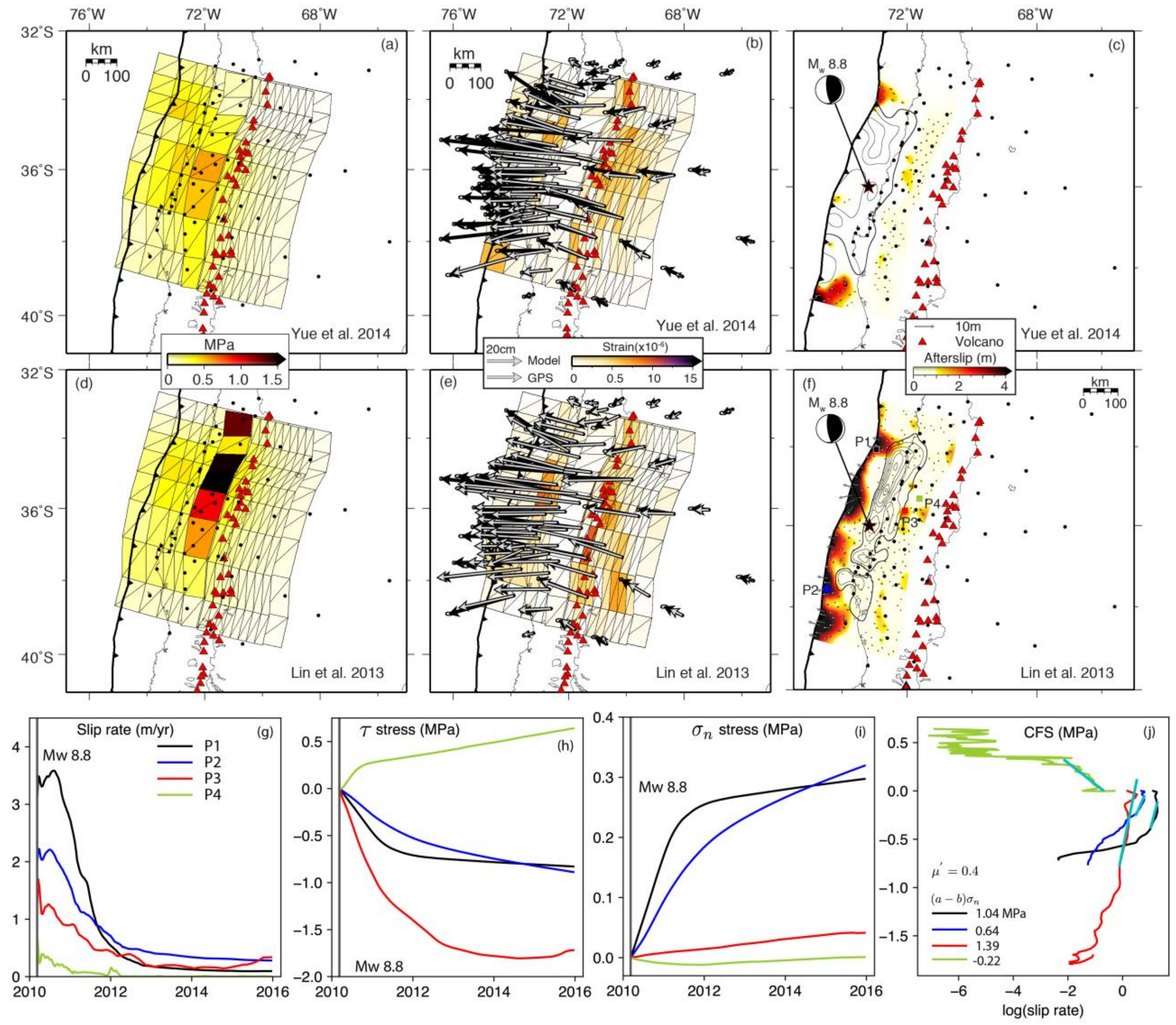

Fig. S7. Afterslip-related test and parameters for fault friction estimates. (**a-f**) Coseismic slip model effects on the inverted afterslip and viscous strain. (a) Coseismic stress changes (second invariant stress tensor), (b) viscous strain and surface displacements, and (c) afterslip estimate from the shallow coseismic slip model (*35*). (d-f) Same as (a-b) but using the slip model employed in the inversion (*24*). (**g-j**) Time evolution of slip rate (g), shear stress (h), normal stress (i) and the relationship between Coulomb stress changes and slip rate (j) for four example triangular fault patches P1-P4. Patch locations are show in Fig. 5 of the main text and fig. S8F. The bold cyan lines in (j) are the best-fit for 2 weeks to 10 months after the earthquake and represent the fault friction parameter *(a-b)*σ_n shown in Fig. 5 of the main text. See Hsu, Simons, Avouac, Galetzka, Sieh, Chlieh, Natawidjaja, Prawirodirdjo and Bock (*4*) for more information on this approach.

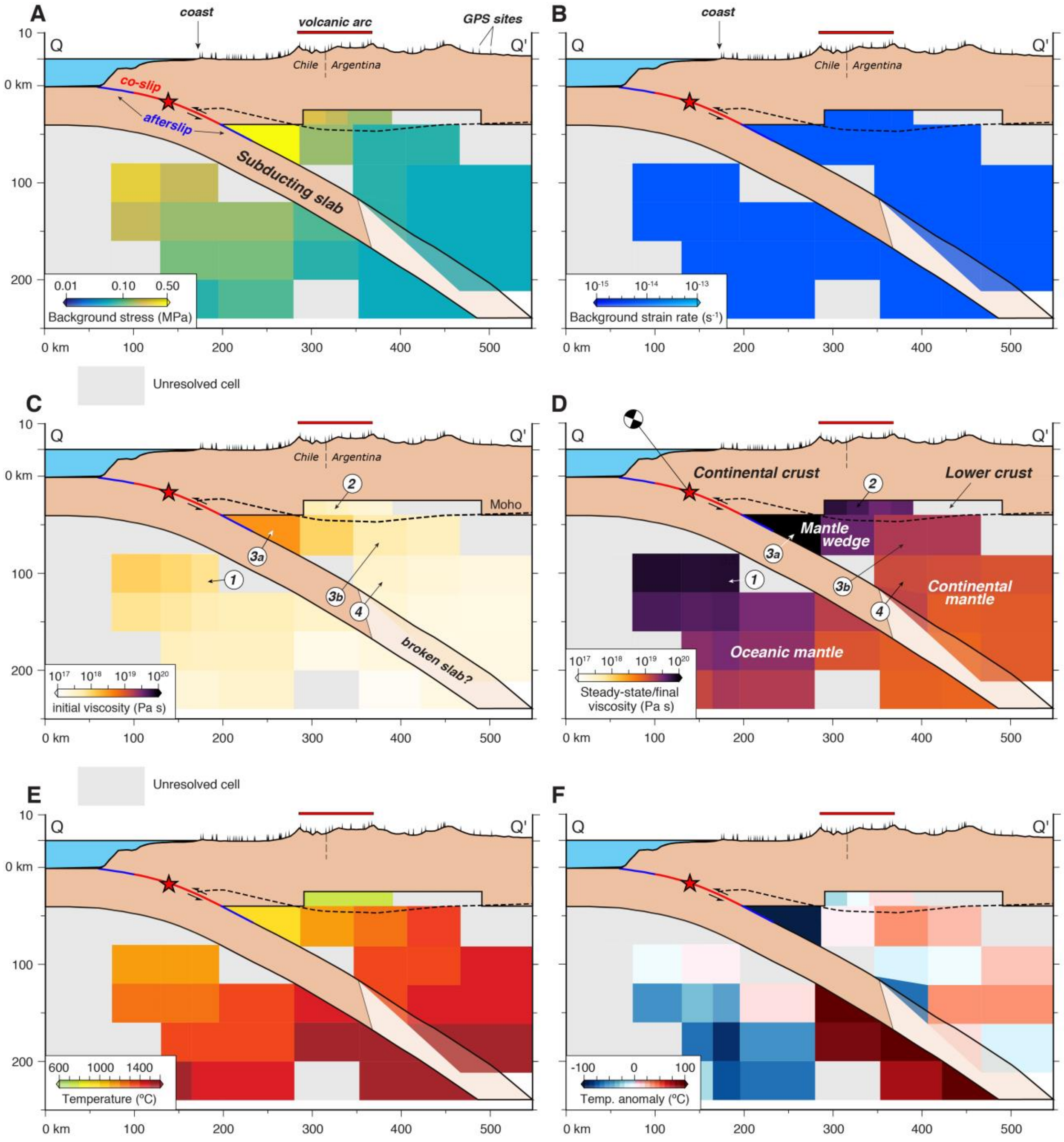

Fig. S8. Cross sections of derived rheological and thermal parameters from inversion and flow law modeling. (A) Background stress (i.e. prestress), (B) background strain rate, (C) initial viscosity, (D) steady-state/final viscosity, (E) temperature, and (F) temperature anomaly for the profile Q-Q'. Profile location shown in Fig. 2 of the main text. Panels (A), (D), and (E) are identical to those shown in Fig. 5 of the main text. The temperature anomaly shown in (F) is the difference from the depth-averaged mean temperature and highlights prominent features in the thermal model. Black dashed line is the continental Moho (*67*).

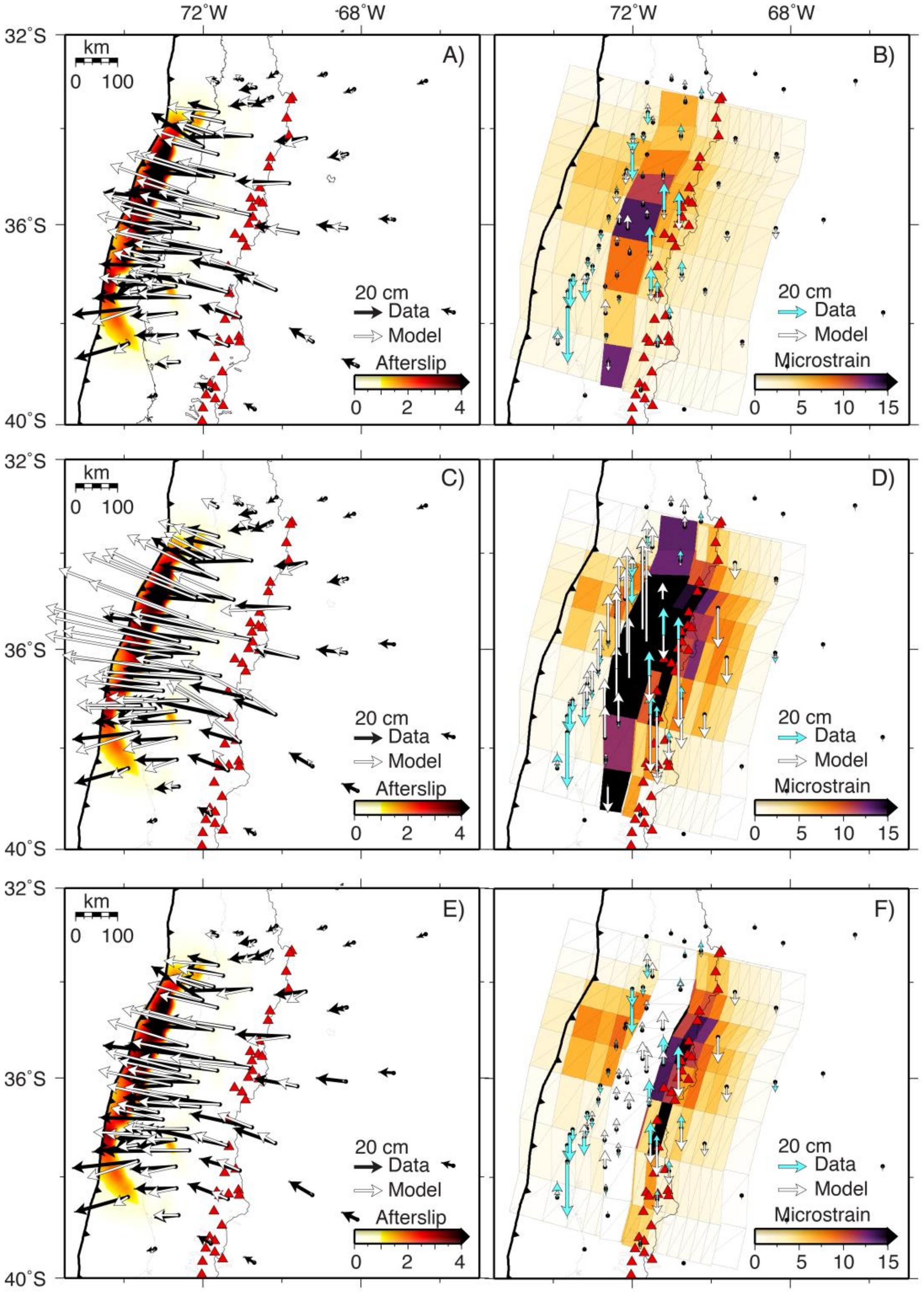

Fig. S9. 3D, stress-driven, postseismic forward models of frictional afterslip and viscoelstic flow for comparison with inversion results. Afterslip (left) and viscous strain (right) for UniCyclE models with a (**a**-**b**) Burgers rheology, (**c**-**d**) nonlinear, power-law rheology, and (**e**-**f**) non-linear, power-law rheology with penalized strain in the mantle wedge. Red triangles are Holocene volcanoes. See Supplementary Materials text for model details.

Table S1. Dislocation creep rheological parameter estimates for the Maule region.*

Oceanic mantle		Continental lithosphere-asthenosphere	
		**Lower crust**	**Continental mantle**
**Stress exponent *n***	3.5	3	3.5
**Activation energy *Q* (kJ mol^{-1})**	480	347	465
**Activation volume *V* (m^3/mol)**	$11x10^{-6}$	$11x10^{-6}$	$11x10^{-6}$
**Basal Temperature *T* (°C)**	1380	1360	1360
**Water concentration C_{OH} (H/10^6Si)**	1000	1200	1200
**Water concentration exponent *r***	1.2	1.2	1.2
**Pre-exponential factor *A* ($MPa^{-n}s^{-1}$)**	90	90	90
**Background strain rate (s^{-1})**	10^{-14}	10^{-14}	10^{-14}

*To obtain the optimized dislocation creep parameters listed above we start with values reported in Karato and Jung (2003). Specifically n=3.0±0.1, Q= 510±30 kJ/mol and 470±40 kJ/mol for dry and wet olivine, respectively, V=$11x10^{-6}$ m^3/mol; C_{OH} = 1000 H/10^6Si, r=1.2±0.05, A=90 $MPa^{-n}s^{-1}$, and strain rate=10^{-14} s^{-1}.

Table S2. Temperature estimates for the polyhedra. Unresolved polyhedra are indicated with NaN.

Polyhedra index	Longitude	Latitude	Depth (km)	Temperature (°C)	Temperature uncertainty (1σ)
1	-69.686	-39.341	172.45	NaN	NaN
2	-69.533	-38.408	176.05	1837.9	10.64
3	-69.327	-37.349	182.22	1829.31	5.83
4	-69.019	-36.430	186.73	1818.47	4.72
5	-68.729	-35.720	186.25	NaN	NaN
6	-68.402	-35.154	186.36	NaN	NaN
7	-68.154	-34.636	185.7	NaN	NaN
8	-68.059	-34.066	183.93	NaN	NaN
9	-69.686	-39.341	127.45	1791.29	10.75
10	-69.533	-38.408	131.05	1782.83	7.11
11	-69.327	-37.349	137.22	1739.38	11.14
12	-69.019	-36.430	141.73	1758.33	12.49
13	-68.729	-35.720	141.25	1736.7	10.55
14	-68.402	-35.154	141.36	NaN	NaN
15	-68.154	-34.636	140.7	NaN	NaN
16	-68.059	-34.066	138.93	NaN	NaN
17	-70.477	-39.201	127.45	1770.08	7.89
18	-70.314	-38.269	131.05	1749.92	10.62
19	-70.097	-37.210	137.22	1708.71	8.65
20	-69.780	-36.293	141.73	1759.69	17.6
21	-69.484	-35.585	141.25	1739.14	38.37
22	-69.152	-35.020	141.36	NaN	NaN
23	-68.900	-34.504	140.7	NaN	NaN
24	-68.800	-33.934	138.93	NaN	NaN
25	-69.686	-39.341	87.45	NaN	NaN
26	-69.533	-38.408	91.05	1675.5	9.57
27	-69.327	-37.349	97.22	1662.52	9.97
28	-69.019	-36.430	101.73	1686.68	6.57
29	-68.729	-35.720	101.25	1668.51	17.82
30	-68.402	-35.154	101.36	NaN	NaN
31	-68.154	-34.636	100.7	NaN	NaN
32	-68.059	-34.066	98.93	NaN	NaN
33	-70.477	-39.201	87.45	NaN	NaN
34	-70.314	-38.269	91.05	NaN	NaN
35	-70.097	-37.210	97.22	NaN	NaN
36	-69.780	-36.293	101.73	1665.04	18.1
37	-69.484	-35.585	101.25	1667.95	34.7
38	-69.152	-35.020	101.36	NaN	NaN
39	-68.900	-34.504	100.7	NaN	NaN
40	-68.800	-33.934	98.93	NaN	NaN
41	-71.153	-39.077	87.34	NaN	NaN
42	-70.981	-38.145	90.12	NaN	NaN
43	-70.755	-37.087	95.13	1571.65	29.18
44	-70.430	-36.171	99.39	1646.1	17.47

Table S2 cont.

45	-70.129	-35.465	98.13	1621.24	25.17
46	-69.793	-34.901	97.57	NaN	NaN
47	-69.537	-34.386	97.56	NaN	NaN
48	-69.433	-33.817	96.63	NaN	NaN
49	-70.364	-39.222	164.12	1835.11	4.79
50	-70.203	-38.289	167.71	1788.49	7.73
51	-69.987	-37.230	173.88	1798.85	6.32
52	-69.671	-36.313	178.39	1842.48	6.49
53	-69.377	-35.605	177.92	1808.78	11.97
54	-69.045	-35.039	178.02	NaN	NaN
55	-68.794	-34.523	177.36	NaN	NaN
56	-68.694	-33.953	175.6	NaN	NaN
57	-71.040	-39.098	120.65	1727.39	15.5
58	-70.870	-38.166	123.31	NaN	NaN
59	-70.645	-37.108	127.8	NaN	NaN
60	-70.322	-36.192	131.95	1662.54	8.79
61	-70.022	-35.485	130.56	1662.81	13.9
62	-69.686	-34.921	129.6	NaN	NaN
63	-69.431	-34.406	129.56	NaN	NaN
64	-69.327	-33.836	128.35	NaN	NaN
65	-71.714	-38.970	80.64	NaN	NaN
66	-71.535	-38.039	83.01	NaN	NaN
67	-71.301	-36.982	87.75	NaN	NaN
68	-70.971	-36.068	91.93	NaN	NaN
69	-70.665	-35.362	90.3	NaN	NaN
70	-70.326	-34.800	89.68	NaN	NaN
71	-70.067	-34.286	89.98	NaN	NaN
72	-69.959	-33.717	89.33	NaN	NaN
73	-74.573	-38.379	220	NaN	NaN
74	-74.446	-37.433	220	NaN	NaN
75	-74.320	-36.350	220	NaN	NaN
76	-74.068	-35.419	220	NaN	NaN
77	-73.831	-34.701	220	NaN	NaN
78	-73.639	-34.111	220	NaN	NaN
79	-73.469	-33.580	220	NaN	NaN
80	-73.293	-33.023	220	NaN	NaN
81	-73.856	-38.535	220	1803.91	18.54
82	-73.737	-37.588	220	1767.28	18.14
83	-73.621	-36.504	220	1746.26	20.69
84	-73.377	-35.572	220	1700.78	23.76
85	-73.145	-34.853	220	1667.86	24.83
86	-72.957	-34.261	220	NaN	NaN
87	-72.791	-33.729	220	NaN	NaN
88	-72.619	-33.172	220	NaN	NaN
89	-73.357	-38.640	220	1787.66	11.43
90	-73.245	-37.693	220	1744.04	20.16
91	-73.135	-36.609	220	1719.77	25.78
92	-72.896	-35.675	220	1704.88	16.2
93	-72.668	-34.955	220	1694.3	18.43

Table S2 cont.

94	-72.484	-34.363	220	1697.19	18.24
95	-72.320	-33.830	220	1710.34	15.32
96	-72.151	-33.272	220	1734.79	15.32
97	-72.996	-38.715	220	NaN	NaN
98	-72.888	-37.768	220	1745.34	23.15
99	-72.784	-36.683	220	1660.76	24.34
100	-72.549	-35.749	220	1655.33	15.46
101	-72.324	-35.028	220	1634.49	16.23
102	-72.141	-34.435	220	NaN	NaN
103	-71.980	-33.902	220	NaN	NaN
104	-71.813	-33.344	220	NaN	NaN
105	-72.393	-38.837	220	NaN	NaN
106	-72.257	-37.896	220	NaN	NaN
107	-72.151	-36.813	220	NaN	NaN
108	-71.933	-35.876	220	1668.16	22.36
109	-71.713	-35.154	220	1651.18	25.56
110	-71.535	-34.560	220	1652.18	28.99
111	-71.377	-34.027	220	1748.53	30.07
112	-71.214	-33.468	220	NaN	NaN
113	-71.622	-38.988	220	NaN	NaN
114	-71.438	-38.058	220	NaN	NaN
115	-71.276	-36.987	220	NaN	NaN
116	-71.083	-36.046	220	NaN	NaN
117	-70.884	-35.320	220	NaN	NaN
118	-70.711	-34.725	220	1679.38	22.11
119	-70.558	-34.191	220	1679.31	16.92
120	-70.400	-33.631	220	NaN	NaN
121	-70.933	-39.118	220	NaN	NaN
122	-70.749	-38.188	220	NaN	NaN
123	-70.516	-37.132	220	NaN	NaN
124	-70.312	-36.194	220	1798.26	23.44
125	-70.142	-35.463	220	NaN	NaN
126	-69.975	-34.867	220	NaN	NaN
127	-69.826	-34.332	220	1760.22	7.95
128	-69.672	-33.771	220	NaN	NaN
129	-74.573	-38.379	180	NaN	NaN
130	-74.446	-37.433	180	NaN	NaN
131	-74.320	-36.350	180	NaN	NaN
132	-74.068	-35.419	180	NaN	NaN
133	-73.831	-34.701	180	NaN	NaN
134	-73.639	-34.111	180	NaN	NaN
135	-73.469	-33.580	180	NaN	NaN
136	-73.293	-33.023	180	NaN	NaN
137	-73.856	-38.535	180	1800.73	15.6
138	-73.737	-37.588	180	1691.85	15.91
139	-73.621	-36.504	180	1674.37	22.13
140	-73.377	-35.572	180	NaN	NaN
141	-73.145	-34.853	180	1595.53	24.02
142	-72.957	-34.261	180	NaN	NaN

Table S2 cont.

143	-72.791	-33.729	180	NaN	NaN
144	-72.619	-33.172	180	NaN	NaN
145	-73.357	-38.640	180	1771.06	10.09
146	-73.245	-37.693	180	1649.93	20.16
147	-73.135	-36.609	180	1644.31	33.38
148	-72.896	-35.675	180	1608.89	14.43
149	-72.668	-34.955	180	1617.44	17.96
150	-72.484	-34.363	180	1625.58	20.97
151	-72.320	-33.830	180	1711.61	22
152	-72.151	-33.272	180	1756.82	19.51
153	-72.996	-38.715	180	1835.58	10.03
154	-72.888	-37.768	180	NaN	NaN
155	-72.784	-36.683	180	1581.08	28.78
156	-72.549	-35.749	180	1590.23	13.44
157	-72.324	-35.028	180	1573.27	16.35
158	-72.141	-34.435	180	1576.05	14.56
159	-71.980	-33.902	180	1646.79	10.29
160	-71.813	-33.344	180	NaN	NaN
161	-72.393	-38.837	180	NaN	NaN
162	-72.257	-37.896	180	NaN	NaN
163	-72.151	-36.813	180	NaN	NaN
164	-71.933	-35.876	180	1620.71	15.93
165	-71.713	-35.154	180	1572.09	16.28
166	-71.535	-34.560	180	1573.61	11.29
167	-71.377	-34.027	180	1697.05	7.34
168	-71.214	-33.468	180	1817.72	10.16
169	-71.622	-38.988	180	NaN	NaN
170	-71.438	-38.058	180	NaN	NaN
171	-71.276	-36.987	180	NaN	NaN
172	-71.083	-36.046	180	1759.18	12.07
173	-70.884	-35.320	180	NaN	NaN
174	-70.711	-34.725	180	1648.19	32.95
175	-70.558	-34.191	180	1642.77	23.95
176	-70.400	-33.631	180	1746.83	28.47
177	-74.573	-38.379	140	NaN	NaN
178	-74.446	-37.433	140	NaN	NaN
179	-74.320	-36.350	140	NaN	NaN
180	-74.068	-35.419	140	NaN	NaN
181	-73.831	-34.701	140	NaN	NaN
182	-73.639	-34.111	140	NaN	NaN
183	-73.469	-33.580	140	NaN	NaN
184	-73.293	-33.023	140	NaN	NaN
185	-73.856	-38.535	140	1777.13	17.47
186	-73.737	-37.588	140	1587.9	10.01
187	-73.621	-36.504	140	NaN	NaN
188	-73.377	-35.572	140	1526.74	17.81
189	-73.145	-34.853	140	1527.29	17.33
190	-72.957	-34.261	140	NaN	NaN
191	-72.791	-33.729	140	NaN	NaN

Table S2 cont.

192	-72.619	-33.172	140	NaN	NaN
193	-73.357	-38.640	140	1771.58	17.81
194	-73.245	-37.693	140	1569.85	14.66
195	-73.135	-36.609	140	1578.71	20.51
196	-72.896	-35.675	140	1541.78	24.92
197	-72.668	-34.955	140	1540.7	13.36
198	-72.484	-34.363	140	1558.98	22.14
199	-72.320	-33.830	140	1641.75	28.76
200	-72.151	-33.272	140	1751.88	17.26
201	-72.996	-38.715	140	NaN	NaN
202	-72.888	-37.768	140	1625.71	14.19
203	-72.784	-36.683	140	1541.51	18.67
204	-72.549	-35.749	140	1536.69	29.01
205	-72.324	-35.028	140	1518.72	13.5
206	-72.141	-34.435	140	1547.09	31.11
207	-71.980	-33.902	140	1623.99	28.29
208	-71.813	-33.344	140	NaN	NaN
209	-72.393	-38.837	140	NaN	NaN
210	-72.257	-37.896	140	NaN	NaN
211	-72.151	-36.813	140	NaN	NaN
212	-71.933	-35.876	140	1590.23	22.99
213	-71.713	-35.154	140	NaN	NaN
214	-71.535	-34.560	140	1525.04	39.81
215	-71.377	-34.027	140	1633.82	34.09
216	-71.214	-33.468	140	NaN	NaN
217	-74.573	-38.379	100	NaN	NaN
218	-74.446	-37.433	100	NaN	NaN
219	-74.320	-36.350	100	NaN	NaN
220	-74.068	-35.419	100	NaN	NaN
221	-73.831	-34.701	100	NaN	NaN
222	-73.639	-34.111	100	NaN	NaN
223	-73.469	-33.580	100	NaN	NaN
224	-73.293	-33.023	100	NaN	NaN
225	-73.856	-38.535	100	1696.53	22.33
226	-73.737	-37.588	100	1515.56	11.1
227	-73.621	-36.504	100	1516.04	11.7
228	-73.377	-35.572	100	1472.28	22.74
229	-73.145	-34.853	100	1451.47	22.12
230	-72.957	-34.261	100	1541.71	25.5
231	-72.791	-33.729	100	NaN	NaN
232	-72.619	-33.172	100	NaN	NaN
233	-73.357	-38.640	100	1683.39	19.71
234	-73.245	-37.693	100	1530.63	10.97
235	-73.135	-36.609	100	1547.81	11.69
236	-72.896	-35.675	100	1481.03	23.48
237	-72.668	-34.955	100	1456.36	15.38
238	-72.484	-34.363	100	1475.11	18.17
239	-72.320	-33.830	100	NaN	NaN
240	-72.151	-33.272	100	NaN	NaN

Table S2 cont.

241	-72.996	-38.715	100	NaN	NaN
242	-72.888	-37.768	100	1596.89	8.45
243	-72.784	-36.683	100	1522.69	9.49
244	-72.549	-35.749	100	1481.93	27.63
245	-72.324	-35.028	100	1436.92	12.11
246	-72.141	-34.435	100	1499.39	23.94
247	-71.980	-33.902	100	NaN	NaN
248	-71.813	-33.344	100	NaN	NaN
249	-70.338	-39.227	226.67	NaN	NaN
250	-70.168	-38.295	226.67	NaN	NaN
251	-69.922	-37.242	226.67	1775.19	13.45
252	-69.708	-36.306	226.67	1817.78	6.88
253	-69.553	-35.573	226.67	1785.92	3.76
254	-69.389	-34.976	226.67	1764.84	2.77
255	-69.244	-34.441	226.67	NaN	NaN
256	-69.094	-33.880	226.67	1776.65	2.17
257	-71.050	-39.096	186.67	NaN	NaN
258	-70.862	-38.167	186.67	NaN	NaN
259	-70.634	-37.110	186.67	NaN	NaN
260	-70.437	-36.170	186.67	1785.74	16.84
261	-70.263	-35.440	186.67	1758.98	5.99
262	-70.095	-34.844	186.67	NaN	NaN
263	-69.946	-34.309	186.67	NaN	NaN
264	-69.791	-33.749	186.67	NaN	NaN
265	-71.735	-38.966	146.67	NaN	NaN
266	-71.554	-38.035	146.67	NaN	NaN
267	-71.408	-36.961	146.67	NaN	NaN
268	-71.216	-36.020	146.67	1686.8	6.38
269	-71.009	-35.295	146.67	NaN	NaN
270	-70.836	-34.701	146.67	NaN	NaN
271	-70.682	-34.166	146.67	NaN	NaN
272	-70.523	-33.606	146.67	NaN	NaN
273	-72.538	-38.808	106.67	NaN	NaN
274	-72.410	-37.865	106.67	NaN	NaN
275	-72.309	-36.781	106.67	NaN	NaN
276	-72.085	-35.845	106.67	NaN	NaN
277	-71.864	-35.123	106.67	NaN	NaN
278	-71.685	-34.530	106.67	1443.09	14.69
279	-71.526	-33.996	106.67	1522.31	9.21
280	-71.361	-33.437	106.67	1623.5	3.79
281	-70.023	-39.331	32.5	NaN	NaN
282	-69.865	-38.397	32.5	NaN	NaN
283	-69.654	-37.333	32.5	NaN	NaN
284	-69.340	-36.413	32.5	NaN	NaN
285	-69.091	-35.761	32.5	NaN	NaN
286	-68.798	-35.259	32.5	NaN	NaN
287	-68.509	-34.759	32.5	NaN	NaN
288	-68.376	-34.131	32.5	NaN	NaN
289	-70.306	-39.281	32.5	NaN	NaN

Table S2 cont.

290	-70.144	-38.348	32.5	NaN	NaN
291	-69.929	-37.284	32.5	NaN	NaN
292	-69.612	-36.364	32.5	NaN	NaN
293	-69.361	-35.712	32.5	NaN	NaN
294	-69.066	-35.211	32.5	NaN	NaN
295	-68.775	-34.711	32.5	NaN	NaN
296	-68.641	-34.084	32.5	NaN	NaN
297	-70.588	-39.230	32.5	NaN	NaN
298	-70.423	-38.297	32.5	NaN	NaN
299	-70.204	-37.234	32.5	NaN	NaN
300	-69.884	-36.314	32.5	NaN	NaN
301	-69.631	-35.663	32.5	NaN	NaN
302	-69.334	-35.162	32.5	NaN	NaN
303	-69.042	-34.663	32.5	NaN	NaN
304	-68.906	-34.036	32.5	NaN	NaN
305	-70.870	-39.179	32.5	NaN	NaN
306	-70.701	-38.246	32.5	NaN	NaN
307	-70.478	-37.183	32.5	NaN	NaN
308	-70.155	-36.264	32.5	NaN	NaN
309	-69.900	-35.613	32.5	NaN	NaN
310	-69.602	-35.113	32.5	NaN	NaN
311	-69.308	-34.615	32.5	NaN	NaN
312	-69.170	-33.987	32.5	NaN	NaN
313	-71.151	-39.126	32.5	NaN	NaN
314	-70.979	-38.194	32.5	NaN	NaN
315	-70.752	-37.131	32.5	1048.34	16.23
316	-70.426	-36.213	32.5	1046.07	28.31
317	-70.169	-35.563	32.5	NaN	NaN
318	-69.869	-35.063	32.5	NaN	NaN
319	-69.574	-34.565	32.5	NaN	NaN
320	-69.434	-33.938	32.5	NaN	NaN
321	-71.432	-39.073	32.5	NaN	NaN
322	-71.256	-38.141	32.5	1098.63	52.59
323	-71.025	-37.079	32.5	1044.56	41.44
324	-70.697	-36.161	32.5	1053	57.94
325	-70.437	-35.512	32.5	1050.64	75.03
326	-70.136	-35.012	32.5	1038.61	61.5
327	-69.840	-34.515	32.5	NaN	NaN
328	-69.698	-33.888	32.5	NaN	NaN
329	-71.712	-39.020	32.5	NaN	NaN
330	-71.533	-38.088	32.5	1070.37	6.12
331	-71.299	-37.026	32.5	1027.92	20.67
332	-70.967	-36.109	32.5	1032.05	25.53
333	-70.705	-35.460	32.5	1034.57	145.46
334	-70.403	-34.961	32.5	1026.03	60.8
335	-70.105	-34.465	32.5	1059.27	64.95
336	-69.961	-33.838	32.5	NaN	NaN
337	-71.992	-38.965	32.5	NaN	NaN
338	-71.810	-38.034	32.5	NaN	NaN

Table S2 cont.

339	-71.571	-36.973	32.5	1015.73	41.06
340	-71.237	-36.056	32.5	1005.53	42.87
341	-70.973	-35.408	32.5	1002.7	114.36
342	-70.669	-34.910	32.5	992.03	47.38
343	-70.370	-34.414	32.5	1030.91	38.52
344	-70.224	-33.787	32.5	NaN	NaN
345	-72.359	-38.860	49.05	NaN	NaN
346	-72.223	-37.920	50.27	NaN	NaN
347	-72.031	-36.852	52.3	NaN	NaN
348	-71.723	-35.932	53.8	1260.8	9.45
349	-71.406	-35.246	53.67	NaN	NaN
350	-71.077	-34.711	53.68	NaN	NaN
351	-70.823	-34.200	53.42	1342.39	11.09
352	-70.691	-33.620	52.87	1241.27	3.99
353	-69.686	-39.341	53.72	NaN	NaN
354	-69.533	-38.408	55.52	NaN	NaN
355	-69.327	-37.349	58.61	NaN	NaN
356	-69.019	-36.430	60.86	NaN	NaN
357	-68.729	-35.720	60.63	NaN	NaN
358	-68.402	-35.154	60.68	NaN	NaN
359	-68.154	-34.636	60.35	NaN	NaN
360	-68.059	-34.066	59.47	NaN	NaN
361	-70.477	-39.201	53.72	NaN	NaN
362	-70.314	-38.269	55.52	NaN	NaN
363	-70.097	-37.210	58.61	NaN	NaN
364	-69.780	-36.293	60.86	1550.93	5.56
365	-69.484	-35.585	60.63	1541.82	4.38
366	-69.152	-35.020	60.68	NaN	NaN
367	-68.900	-34.504	60.35	NaN	NaN
368	-68.800	-33.934	59.47	NaN	NaN
369	-71.153	-39.077	53.72	NaN	NaN
370	-70.981	-38.145	55.52	1566.68	3.15
371	-70.755	-37.087	58.61	1522.72	2.49
372	-70.430	-36.171	60.86	1555.2	3.91
373	-70.129	-35.465	60.63	1512.46	4.19
374	-69.793	-34.901	60.68	NaN	NaN
375	-69.537	-34.386	60.54	NaN	NaN
376	-69.433	-33.817	60.07	NaN	NaN
377	-71.826	-38.949	53.72	NaN	NaN
378	-71.645	-38.017	55.52	1480.21	12.03
379	-71.410	-36.961	58.61	1438.8	4.33
380	-71.079	-36.047	60.86	1468.16	4.12
381	-70.772	-35.342	60.63	1436.77	6.63
382	-70.432	-34.780	60.68	1432.97	3.85
383	-70.173	-34.266	60.58	1489.25	3.01
384	-70.064	-33.696	60.12	NaN	NaN
